# Chalcone-based Selective Inhibitors of a C_4_ Plant Key Enzyme as Novel Potential Herbicides

**DOI:** 10.1038/srep27333

**Published:** 2016-06-06

**Authors:** G. T. T. Nguyen, G. Erlenkamp, O. Jäck, A. Küberl, M. Bott, F. Fiorani, H. Gohlke, G. Groth

**Affiliations:** 1 Biochemical Plant Physiology, Heinrich Heine University Düsseldorf and Bioeconomy Science Center (BioSC), Universitätsstr.1, 40225 Düsseldorf, Germany; 2 Pharmaceutical and Medicinal Chemistry, Heinrich Heine University Düsseldorf and Bioeconomy Science Center (BioSC), Universitätsstr.1, 40225 Düsseldorf, Germany; 3 Institute of Bio- and Geosciences, IBG-2: Plant Sciences, Forschungszentrum Jülich and Bioeconomy Science Center (BioSC), Wilhelm-Johnen-Straße, 52425 Jülich, Germany; 4 Institute of Bio- and Geosciences, IBG-1: Biotechnology, Forschungszentrum Jülich and Bioeconomy Science Center (BioSC), Wilhelm-Johnen-Straße, 52425 Jülich, Germany.

## Abstract

Weeds are a challenge for global food production due to their rapidly evolving resistance against herbicides. We have identified chalcones as selective inhibitors of phosphoenolpyruvate carboxylase (PEPC), a key enzyme for carbon fixation and biomass increase in the C_4_ photosynthetic pathway of many of the world’s most damaging weeds. In contrast, many of the most important crop plants use C_3_ photosynthesis. Here, we show that 2′,3′,4′,3,4-Pentahydroxychalcone (IC_50_ = 600 nM) and 2′,3′,4′-Trihydroxychalcone (IC_50_ = 4.2 μM) are potent inhibitors of C_4_ PEPC but do not affect C_3_ PEPC at a same concentration range (selectivity factor: 15–45). Binding and modeling studies indicate that the active compounds bind at the same site as malate/aspartate, the natural feedback inhibitors of the C_4_ pathway. At the whole plant level, both substances showed pronounced growth-inhibitory effects on the C_4_ weed *Amaranthus retroflexus*, while there were no measurable effects on oilseed rape, a C_3_ plant. Growth of selected soil bacteria was not affected by these substances. Our chalcone compounds are the most potent and selective C_4_ PEPC inhibitors known to date. They offer a novel approach to combat C_4_ weeds based on a hitherto unexplored mode of allosteric inhibition of a C_4_ plant key enzyme.

Worldwide, weeds cause more yield loss and add more to farmers’ production costs than any other agricultural pest. This weed challenge on global food production has become severe due to rapidly evolving resistance of many weed species, resulting in resistance against herbicides addressing 22 of the 25 molecular targets known to date for weed control. Many of the worst weeds use C_4_ photosynthesis, whereas the majority of crop plant species use the classical C_3_ photosynthetic pathway. Hence, enzymes of the C_4_ pathway provide an excellent target to combat these weeds. In C_4_ photosynthesis, inorganic carbon is initially fixed by the enzyme phosphoenolpyruvate (PEP) carboxylase (PEPC), yielding the four-carbon molecule oxaloacetate^1^. Oxaloacetate is then reduced to malate or transaminated to aspartate in the decarboxylation reaction of the C_4_ pathway^2^. There is a major difference in the sensitivity of PEPC from C_3_ and C_4_ plants towards feedback inhibition by C_4_ carboxylates from the C_4_ pathway^3^. Previously, we selected the genus *Flaveria* as a model to study the feedback inhibitor tolerance of PEPC of C_3_ and C_4_ plants^4^ because this genus contains various C_3_, C_3_-C_4_ intermediate, and C_4_ species^5^. A single residue in the dicarboxylate feedback inhibitor binding site was shown to control the different malate tolerance of C_3_ and C_4_ plants^4^: Arginine-884 of *F. pringlei* (C_3_ plant) PEPC assists the feedback inhibitor binding, whereas glycine at the same position of *F. trinervia* (C_4_ plant) PEPC forms no interaction with the inhibitor^4^. Arginine-884 is conserved in all typical C_3_ crop plants. In most C_4_ weeds, glycine, serine, or glutamine are found in this position^6^. Hence, the molecular difference in the feedback inhibitor binding site of PEPC in C_3_ and C_4_ plants should allow developing selective herbicides for weed control. We showed that catechins and quinoxalines are selective C_4_ PEPC inhibitors with IC_50_ values in the range of 100 μM^7^. However, small molecule compounds with enhanced inhibitory effects and selectivity for C_4_ PEPC are required to advance further development of C_4_ selective herbicides.

In this study, based on their chemical and structural similarity with the previously introduced C_4_-selective catechine inhibitors^7^, we identify members of the chalcone family from chemical libraries as suitable selective inhibitors for C_4_ PEPC. Effects on plant growth regulation and early development have been reported for *trans*-chalcone for a variety of crop species and associated weeds^8^. However, no molecular target or mode of action was identified in these studies. We applied activity assays and binding studies to elucidate the inhibitory effects of *trans*-chalcone and eleven hydroxyl derivatives on a typical C_3_ and C_4_ PEPC from the genus *Flaveria*. Molecular modeling and simulation studies suggest a binding model of the compounds at the PEPC feedback inhibitor binding site. *In planta* experiments confirmed the inhibitory effects of chalcones on weed growth. We found that the quantity and position of hydroxyl groups influence the potency and selectivity of chalcones on PEPC from *F. pringlei* and *F. trinervia.* In all, our study identifies new lead structures for the development of selective herbicides and highlights a novel mode of action against C_4_ weeds.

## Results

### Chalcones are potent inhibitors of PEPC

Computational screening using the feedback inhibitor binding pockets of C_4_ PEPC from *F. trinervia* (PDB ID 3ZGE) and C_3_ PEPC from *F. pringlei* (PDB ID 3ZGB) as models indicated that the plant polyphenol butein (**8**) could be a potential inhibitor against the C_4_ over the C_3_ isoform. Butein is a chalcone containing two hydroxyl groups on either ring A and B. We chose to test chalcones with different numbers and positions of hydroxyl groups including *trans*-chalcone (**1**) and its related compounds (Table 1 and Fig. S1). *Trans*-chalcone (**1**) containing no hydroxyl group shows weak inhibition of both isoforms (IC_50, C4_ = ~78 μM, IC_50, C3_ = ~120 μM). The inhibitory effect is also weak for other chalcones containing only one hydroxyl group attached to ring A and/or ring B (**2**, **3**). Compounds containing two hydroxyl groups attached to ring A (**4**, **5**) and those with an additional hydroxyl group on ring B (**6**, **7**) inhibit PEPC with IC_50,C4_ = 30–55 μM and IC_50,C3_ = 55–100 μM. The selectivity for C_4_ PEPC compared to C_3_ PEPC of compounds **1**–**7** is low, with a maximal selectivity factor of two (compound **5**). The presence of two hydroxyl groups on each ring (butein, **8**) leads to a distinct inhibitory effect with IC_50,C4_ = 2.2 μM. A similar effect was observed for robtein (**9**), a compound with two hydroxyl groups on ring A and three on ring B, and for 2′,3′,4′‐Trihydroxychalcone (**10**) and 2′,4′,6′,3,4‐Pentahydroxychalcone (**11**), compounds containing three hydroxyl groups on ring A and none or two on ring B, respectively. These results indicate that the number of hydroxyl groups on ring A of the chalcones positively affects their PEPC inhibition potency. Furthermore, if ring A contains only two hydroxyl groups, ring B needs to have at least two hydroxyl groups for a good inhibitory effect. The IC_50,C3_ values of butein (**8**), robtein (**9**), **10** and **11** are ~2.5 μM, whereas the IC_50,C4_ value of okanin (**12**) is 0.6 μM (Table 1). These results indicate that the hydroxyl group at position 3′ of ring A (**10**) or positions 3, 4 of ring B (**8**, **9**, **11**) are necessary to increase inhibition of C_4_ PEPC in comparison to **1–7**. Okanin (**12**) containing hydroxyl groups at both 3′ of ring A and 3, 4 of ring B has a ~5-fold lower IC_50_ value in comparison to **8–11**, implying that not only the number but also the position of hydroxyl groups affects the inhibitory potency. The IC_50_ values of **8**–**12** on C_4_ PEPC are up to 45-fold lower than on C_3_ PEPC showing that these compounds have promising selectivity for C_4_ PEPC, with okanin (**12**) being the most selective compound (Table 1). Compound **11** and okanin (**12**) are regioisomers (Table 1). The hydroxyl group at position 6′ of ring A (**11**) seems to decrease the selectivity for C_4_ vs. C_3_ PEPC.

### Localization of PEPC-chalcone binding site

**Figure 1:**
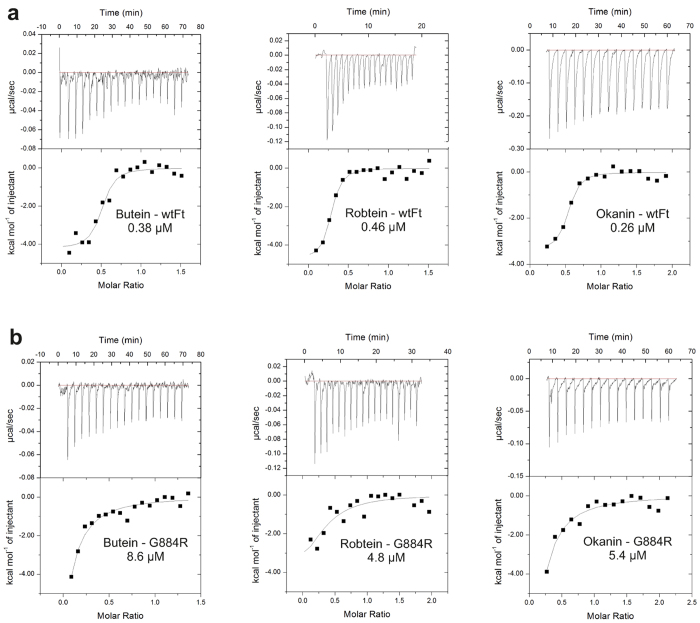
Binding affinities of butein (8), robtein (9), and okanin (12) to C_4_ PEPC from *F. trinervia*. (**a**) ITC binding curves of the compounds binding to the wild-type (wtFt). (**b**) ITC binding curves of the compounds binding to the mutant G884R at lower affinities.

**Figure 2:**
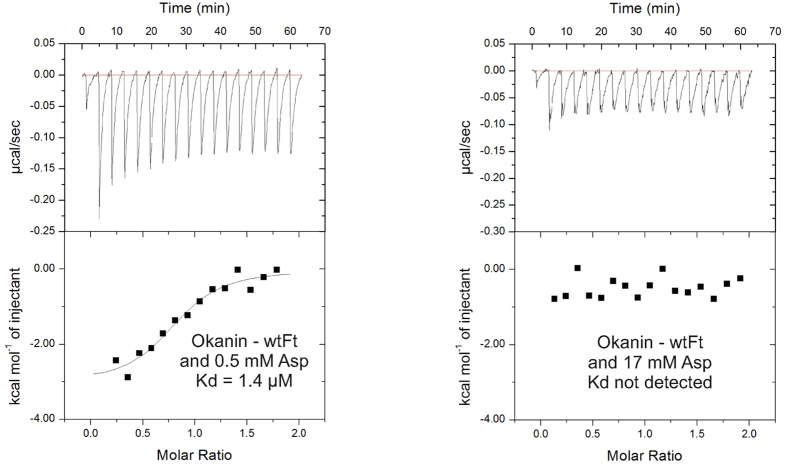
Competition between okanin (12) and the feedback inhibitor aspartate in binding to the wild-type *F. trinervia* PEPC. ITC binding curves of okanin (**12**) binding to C_4_ PEC from *F. trinervia* in the presence of 0.5 mM or 17 mM aspartate.

G884 of C_4_ PEPC (R884 of C_3_ PEPC) is a key residue in the feedback inhibitor binding pocket^4^. To identify the binding site of chalcones, binding affinities to the wild-type C_4_ PEPC containing G884 and to mutant G884R mimicking the corresponding binding site in C_3_ PEPC were measured using ITC. The chalcones butein (**8**), robtein (**9**), and okanin (**12**) have moderate binding affinities to the wild-type C_4_ PEPC with *K*_d_ values of 0.38 μM, 0.46 μM and 0.26 μM, respectively (Fig. 1a). The compounds bind to the mutant G884R with at least 10-fold weaker affinity confirming the selectivity role of residue 884 in PEPC (Fig. 1b). These results are consistent with our data from activity assays. They suggest that the compounds bind to the feedback inhibitor binding pocket. To test whether chalcones bind to C_4_ PEPC at the same site as the feedback inhibitor, okanin (**12**) was titrated against the protein in the presence of aspartate (Fig. 2). At 0.5 mM aspartate, okanin (**12**) can still bind to the enzyme with a *K*_d_ value of 1.4 μM, ~5-fold weaker in comparison to the sample with no aspartate (0.26 μM). In the presence of 17 mM aspartate (2-fold IC_50_ value of the feedback inhibitor on C_4_ PEPC^9^), no binding can be detected implying that aspartate competes with okanin for the same binding site.

### Binding mode model of the chalcones and structure-activity/selectivity relationships

To identify a model of the binding mode of chalcones in the feedback inhibitor binding pocket, molecular docking was applied. The approach was initially validated by redocking aspartate to C_4_ PEPC (PDB ID 3ZGE) and C_3_ PEPC (PDB ID 3ZGB) (Fig. S2a). This yielded lowest energy binding poses with an all-atom root mean square deviation (RMSD) of aspartate to the crystal structures of 0.32 Å (0.25 Å) for C_4_ (C_3_) PEPC (Fig. S2b,c). Next, we docked **1**–**12** (Table 1) to both PEPC variants. This resulted in overall similar binding poses (Fig. S3; mean mutual RMSD of the core atoms 1.27 Å after energy minimization^10^,^11^).

**Figure 3:**
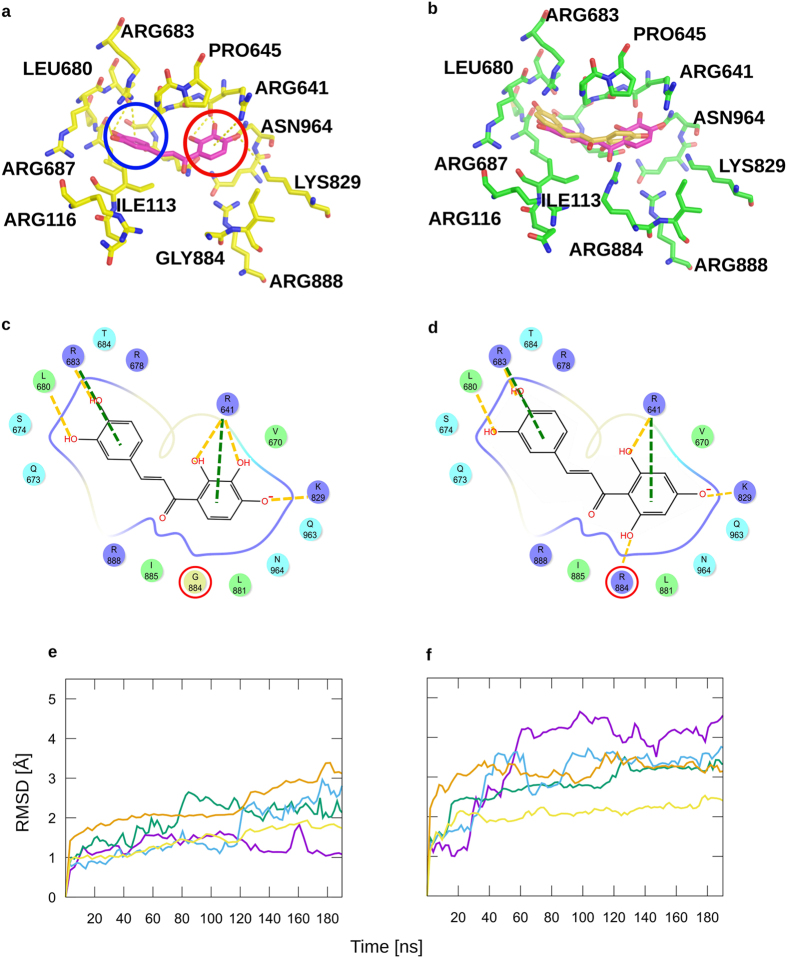
Prediction of the binding mode of chalcones. (**a**) Pose of **12** (magenta) within C_4_ PEPC after MAB minimization of the configuration docked with GLIDE with the lowest energy in the largest cluster. Very similar binding poses are also found for all other chalcones tested here (see Fig. S3). Ring A (red circle) is located in between the two arginines (R641 and R888) that are important for Asp binding. Ring B (blue circle) is deeply buried in a subpocket unused by Asp. (**b**) Starting configuration (magenta) of **12** and configuration after 200 ns of MD simulations (gold) in C_3_ PEPC. During the MD simulations, **12** shifts by ~3 Å RMSD. (**c,d**) 2D schemes of the binding poses of **12** in C_4_ PEPC (**c**) and **11** in C_3_ PEPC (**d**). The red circles mark G884 and R884, which is the selectivity-determining residue. (**e**,**f**) Representative RMSD with respect to the starting structure over the course of three independent MD simulations each of **12** in C_4_ PEPC (**e**) and **12** in C_3_ PEPC (**f**) for the backbone atoms of PEPC (orange) and side-chains atoms of PEPC within 5 Å distance of the starting configuration of the ligand (yellow). The blue, green, and violet lines depict RMSD values of the ligands with respect to the starting configuration for the three independent MD simulations each of a PEPC-ligand complex. For better visibility smoothing was applied for all plots. (**a**,**b**) Ring B is buried inside a subpocket, which is formed by A132, E135, Q673, H679, L680, C681, R683, and R687. For clarity, residues A132, E135, Q673, H679, and C681 are not represented; none of the ligands investigated in this study interact with these amino acids.

As shown for okanin (**12**) **–** the chalcone with the highest affinity and selectivity for C_4_ PEPC - ring A is located close to R641 and R888 (Fig. 3a). These residues engage in cation-π interactions with ring A of the chalcones (Fig. 3a,c; distance guanidino nitrogens center of the phenyl ring: ~3.6 Å for **12**). The hydroxyl group in 2′ position in ring A in okanin (**12**) and **2–11** (Figs S3 and 3a–d, for clarity only okanin (**12**) and **8–10** are shown) forms a hydrogen bond with the carbonyl oxygen of R641. The hydroxyl group in 3′ position of okanin (**12**) forms an additional hydrogen bond with R641. For the hydroxyl group in 4′ position in chalcones **4** and **6–12**, which is closely located to the side chain of K829, the computed mean pKa value in water is 7.9 ± 0.8. Considering an average pH in the plant cytoplasm of ~7.5^12^, it is thus plausible that this hydroxyl group binds in the deprotonated form to PEPC, forming a salt bridge with K829. Overall, this can explain why okanin (**12**) with hydroxyl groups in positions 2′,3′, and 4′ shows the highest inhibitory effect towards C_4_ PEPC. Okanin (**12**) lacks a hydroxyl group in 6′ position leaving R884 in C_3_ PEPC without a hydrogen bond acceptor on the side of okanin (**12**) (Fig. 3b) which leads to an unfavorable binding contribution in C_3_ PEPC and to high selectivity of okanin (**12**) for C_4_ PEPC (Table 1). In contrast, the hydroxyl group in position 6′ of **11** forms a hydrogen bond with R884 in C_3_ PEPC (Fig. 3d), leading to a ~14-fold decreased selectivity factor of **11** compared to okanin (**12**) (Table 1).

Ring B of the chalcones is deeply buried inside a subpocket of the feedback inhibitor binding pocket for which no occupation by a ligand has yet been observed in the available crystal structures (Fig. S2b,c). The phenyl ring forms a cation-π interaction with R683 (distance guanidino nitrogen ^…^ center of the phenyl ring: 3.6 Å), and the hydroxyl groups at positions 3 and 4 as in okanin (**12**) establish hydrogen bonds with the backbone of L680 and the side chain of R683, respectively. Compound **10** has no hydroxyl groups at ring B, which can explain its ~4-fold lower inhibitory effect compared to okanin (**12**).

This qualitative structure-activity relationship (SAR) was substantiated by a quantitative SAR model using the protein-based Adaption of Fields for Molecular Comparison (AFMoC) analysis^13^. See supplemental results for details (Fig. S4).

### Stability of the binding modes investigated by MD simulations

PEPC is a dimer of dimers. The feedback inhibitor binding pocket of one monomer is not affected by another monomer^14^,^15^. Hence, we used only one monomer to test for the stability of the suggested binding mode by MD simulations. Three independent all-atom MD simulations of 200 ns length each were performed for the C_3_ and C_4_ PEPC complexes with okanin (**12**), starting from the docked and minimized binding modes of **12**. Figure 3e,f show RMSD values with respect to the starting structure over the course of the MD simulations. As exemplarily depicted for one MD trajectory, the RMSD values for all backbone atoms of C_4_ PEPC remain <3.5 Å. RMSD values of the binding site region fluctuate around 2 Å and 2.5 Å for the two complexes. Overall, these values are comparable to MD simulations of protein-ligand complexes^16^,^17^. For okanin (**12**), the RMSD with respect to the starting conformation relative to C_4_ PEPC is 1.60 ± 0.01 Å RMSD (mean ± standard error of the mean) across all three trajectories, with one trajectory staying below 2 Å, indicating a stable binding mode (Fig. 3e). In contrast, for okanin (**12**) in C_3_ PEPC, the RMSD is 3.12 ± 0.37 Å across all three trajectories (Fig. 3f), and one trajectory reaches RMSD values >4 Å. Accordingly, the ligand shifts considerably within the binding site (Fig. 3b), in line with the much weaker binding affinity of okanin (**12**) in C_3_ PEPC than in C_4_ PEPC (Table 1).

### Analyses of inhibitory effects *in planta*

**Figure 4:**
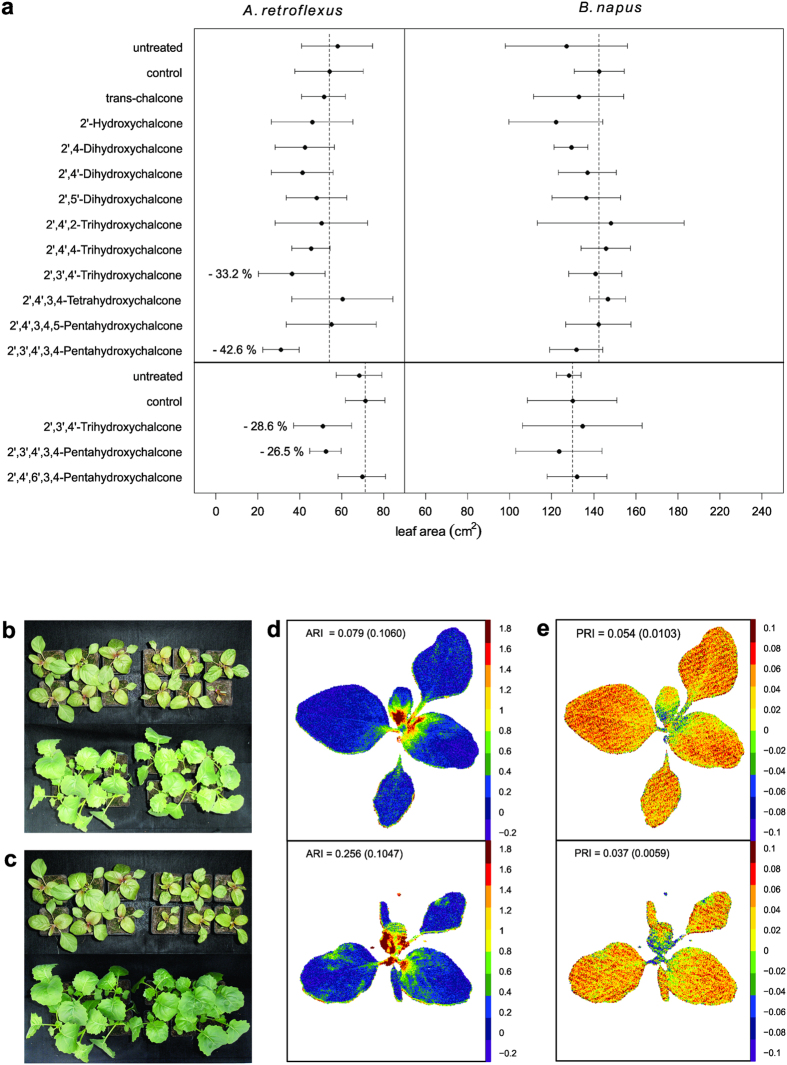
Effects of chalcones on *A. retroflexus* and *B. napus*. (**a**) Leaf area of *A. retroflexus* and *B. napus* six days after treatment. Results of experiment 1 (top) and experiment 2 (bottom) are displayed. 2′,3′,4′-Trihydroxychalcone (**10**) and 2′,3′,4′,3,4-Pentahydroxychalcone (**12**) significantly reduced *A. retroflexus* leaf area in both experiments according to Tukey’s HSD at α = 0.05. None of the compounds had an effect on leaf area of *B. napus*. Bars display standard deviations (n = 6; *B. napus* experiment 2: n = 5). Dashed lines represent the average of the corresponding control treatments. (**b**) Effect of 2′,3′,4′-Trihydroxychalcone (right) on growth of *A. retroflexus* (top) and *B. napus* (bottom) in comparison to the control treatment (left). (**c**) Effect of 2′,3′,4′,3,4-Pentahydroxychalcone (right) on growth of *A. retroflexus* (top) and *B. napus* (bottom) in comparison to the control treatment (left). (**d**) false-color image of anthocyanin reflectance index (ARI) of *A. retroflexus* two days after treatment with 2′,3′,4′,3,4-Pentahydroxychalcone (bottom) and the corresponding control (top). ARI values given inside the image are the average values of the 3^rd^ leaf for six replicates. Standard errors are given in brackets. (**e**) false-color image of photochemical reflectance index (PRI) of *A. retroflexus* two days after treatment with 2′,3′,4′,3,4-Pentahydroxychalcone (bottom) and the corresponding control (top). PRI values given inside the image are the average values of the 3^rd^ leaf for six replicates. Standard errors are given in brackets.

Two of the tested chalcones showed pronounced inhibitory effects on growth of *A. retroflexus* six days after treatment (Fig. 4a) during two independent experiments. In both experiments 2′,3′,4′-trihydroxychalcone (**10**) and okanin (**12**) consistently reduced *A. retroflexus* leaf area ranging on average from about 30% to about 40% compared with control. Furthermore, we ran an ANOVA model combining the two experiments introducing ‘experiment’ as a two-level categorical factor to test whether there was a significant experiment × treatment interaction. This was not the case indicating that the treatment effects were consistent across the two experiments. According to this combined analysis, okanin (**12**) and (**10**) both significantly reduced leaf area according to Tukey’s HSD at α = 0.05 (p < 0.001). Both compounds led to overall reduced growth of *A. retroflexus*, and treated leaves showed deformation of the lamina (Fig. 4b,c). Analysis of leaf area changes of the 2^nd^ leaf (i.e., the youngest treated leaf) of *A. retroflexus* tracked over time revealed a significant reduction of leaf growth caused by **10** and okanin (**12**) (Fig. S5a). Okanin (**12**) and **10** showed similar and significant effects (p = 0.001; Tukey’s HSD at α = 0.05) on leaf biomass formation in a combined analysis of both experiments (experiment x treatment interaction not significant; Fig. S5b). All other tested chalcones did not affect growth of *A. retroflexus* shoots and leaves significantly (Table 1, Figs 4a and S5b). These effects on *A. retroflexus* growth were transient because thirteen days after treatment plants recovered and did not show other visible or measurable damage (*p* = 0.21 and *p* = 0.74 for leaf area of experiment 1 and 2, respectively; *p* = 0.41 and *p* = 0.37 for biomass of experiment 1 and 2, respectively.). We note that none of the tested chalcones affected growth of *B. napus* at either time point indicating selectivity for C_4_ metabolism over C_3_ metabolism during two independent experiments (*p* = 0.33 and 0.90; *p* = 0.98 and 0.29 at six and thirteen days after treatment, respectively, for leaf area; *p* = 0.88 and 0.85; *p* = 0.46 and 0.29 at six and thirteen days after treatment, respectively, for biomass) (Figs 4a and S5b).

Leaf gas exchange measurements in *A. retroflexus* revealed a significant reduction of the maximal PEP carboxylation rate (V_pmax_) one day after treatment with okanin (**12**), which is revealed by reduced CO_2_ assimilation at low CO_2_ partial pressure. V_pmax_ decreased by more than 50% from 36.5 μmol m^−2 ^s^−1^ to 17.2 μmol m^−2 ^s^−1^, while there was no change in V_pmax_ of the control plants (Table 2 and Fig. S6a–d). In *B. napus* leaves there were no changes of photosynthetic parameters after treatment with okanin (**12**). In this experiment there were no pronounced effects on CO_2_ assimilation curves (Fig. S6e–h), however maximal rubisco carboxylation rate (V_cmax_), maximal electron transport rate (J_max_) and mitochondrial respiration (R_d_) showed slight differences between all treatments (Table S3). Control plants that were treated with DMSO revealed decreased V_cmax_ and R_d_ one day after treatment, whereas V_cmax_ increased in untreated plants between the measurement days.

Effects of okanin (**12**) on *A. retroflexus* could be measured in additional experiments by using hyperspectral imaging to quantify effects on photosystems functionality. *A. retroflexus* shoots and leaves treated with okanin (**12**) revealed an altered photochemical reflectance index (PRI) and anthocyanin reflectance index (ARI) compared to control plants (Fig. 4d,e). PRI was significantly reduced in treated plants (*p* = 0.006) whereas ARI was significantly increased (*p* = 0.016), indicating decreased photosystem efficiency. Finally, Chlorophyll fluorescence measurements showed that okanin (**12**) affects PSII photochemistry of *A. retroflexus* (Fig. S7a) but not that of *B. napus* (Fig. S7b). In these experiments okanin (**12**) increased the effective quantum yield of photosystem II, non-photochemical quenching was reduced, and the maximum electron transfer rate was increased. In summary*, in planta* experiments with *A. retroflexus* and *B. napus* indicate selectivity of okanin (**12**) for C_4_ metabolism compared to C_3_ metabolism.

### Influence of chalcone derivatives on the growth of three soil bacteria and *E. coli*

To monitor the effects of the chalcone derivatives acting as potential herbicides on the environment, we tested their effects on the growth of three different species of soil bacteria, namely *Bacillus subtilis* 168, *Corynebacterium glutamicum* ATCC13032, and *Pseudomonas putida* KT2440, and additionally of the gut bacterium *Escherichia coli* K-12 (strain MG1655). For each chalcone, three different concentrations were investigated corresponding to 0.1×, 1×, and 10× of the respective IC_50_ concentration measured for C_4_ PEPC. The growth behavior of the four strains was assessed by the following three parameters: the final cell density (measured as backscatter at 620 nm), the growth rate (h^−1^), and the duration of the lag phase. A summary of the results is shown in Table 1, while a detailed presentation is given in Table S2. The Gram-negative bacteria *E. coli* and *P. putida* did not show growth defects under any condition tested, except for a slight increase in the lag phase of *P. putida* at 400 μM of **4**. In the case of the Gram-positive bacteria *B. subtilis* and *C. glutamicum*, no growth defects were observed at chalcone concentrations below 100 μM (for example, Figs S8 and S9 showing results for **12** and **10**, respectively), except for *C. glutamicum* cultured in the presence of 55 μM of **5** (Fig. S10). In most cases, the addition of the compounds above 100 μM to cultures of *C. glutamicum* and *B. subtilis* led to an extended lag phase or a lower growth rate, but not to a lower cell density. The only exception was **5** at a concentration of 550 μM, which abolished growth of *B. subtilis* and *C. glutamicum* in minimal media.

## Discussion

To date herbicide-resistant weeds have been reported in 66 crops in 61 countries^18^. However, no major herbicide with a new mode of action has been introduced in the last 20 years^19^. Phosphoenolpyruvate carboxylase (PEPC), a key enzyme for carbon fixation and biomass increase in the C_4_ photosynthetic pathway, has been used as a target in studies of C_4_ selectivity and C_4_ photosynthesis^20^. Some compounds such as 3,3-dichloro-2-(dihydroxyphosphinoylmethyl)propenoate and shikimic acid were reported as competitive inhibitors against the PEPC substrate PEP^20^,^21^. However, the selectivity of these compounds on C_4_ over C_3_ plants is not significant. Here, we identified chalcones, in particular 2′,3′,4′,3,4-Pentahydroxychalcone (okanin, **12**), a natural pentachalcone in the *Asteraceae* family^22^ and 2′,3′,4′-Trihydroxychalcone (**10**), as selective inhibitors of C_4_ PEPC. These chalcones offer a novel approach to control C_4_ weeds.

The binding mode of the natural inhibitor aspartate in the crystal structures of PEPCs from C_3_ and C_4_ plants provided the platform for the identification of these chalcones^4^. Binding studies showed that okanin (**12**) is the best C_4_ PEPC inhibitor with an IC_50_ value of 600 nM and a 45-fold selectivity towards C_4_ PEPC over C_3_ PEPC (Table 1); compound **10** shows a seven-fold weaker inhibitory potency and a three-fold lower selectivity (Table 1). These chalcone compounds are the most potent and selective C_4_ PEPC inhibitors known to date^7^,^20^,^21^. Isothermal tritration calorimetry in the presence of the natural dicarboxylate inhibitor aspartate demonstrated that okanine (**12**) binds to the feedback inhibitor binding site. Hence, the chalcone exploits a hitherto unexplored mode of allosteric inhibition of a C_4_ plant key enzyme^4^. The regioisomer of okanine (**12**), 2′,4′,6′,3,4‐Pentahydroxychalcone (**11**) has a ~fourfold weaker inhibitory potency and a ~13-fold lower selectivity. Molecular modeling and simulation studies highlighted the importance of position 6′ in ring A of chalcones for PEPC selectivity in terms of its location in the vicinity of the selectivity-determining residue 884^4^. A small, hydrophobic substituent at this position rather than a hydrophilic group as in **11** may improve the selectivity towards C_4_ PEPC. These studies also provided explanations of the effect of differences in the number and positions of other hydroxyl groups on the potency of the chalcones. Concerning ring A, hydroxyl groups are best located at positions 2′,3′, and 4′. Concerning ring B, which is deeply buried inside a subpocket of the feedback inhibitor binding pocket for which no occupation by a natural ligand has yet been observed, hydroxyl groups are best located at positions 3 and 4.

Application of okanin (**12**) and **10** to seedlings of *A. retroflexus* and *B. napus* indicated efficacy and selectivity for C_4_ metabolism. Growth reduction (leaf area and biomass) of treated shoots and leaves yielded transient and non-systemic effects. These effects resulted from a temporary inhibition of leaf expansion and did not clearly influence the rate of leaf appearance. At the physiological level, we found a significant reduction of the maximal PEP carboxylation rate in *A. retroflexus* by more than 50% after treatment with okanin (**12**). These results are in line with previous experiments showing pronounced effects on assimilation at low CO_2_ concentrations using *Amaranthus edulis* mutants expressing reduced levels of PEPC^23^. In this study an *A. edulis* mutant expressing 55% lower PEPC compared with wild-type also showed a decreased maximal photosynthesis rate. However, mutants with PEPC expression lower than 50% compared with wild-type displayed only minor changes in maximal assimilation rates. In our experiments we did not find any differences in maximal assimilation rates after treatment with okanin (**12**). This lack of measurable effects might be explained by compensation mechanisms as postulated by Dever *et al*.^23^. It is important to note that there was no effect of okanin (12) treatment on CO_2_ assimilation of *B. napus* leaves indicating selectivity of okanin (**12**) for C_4_ photosynthesis. Both electron transfer rate and quantum yield of PSII measured with active fluorescence were increased by treatment of *A. retroflexus* with okanin (**12**). This is apparently in contrast with what would be expected by PEPC-inhibition. A possible explanation is that other metabolic pathways are involved, namely the activation of detoxification driven by NADPH P450 monooxygenases leading to a possible increased demand of reductant and thereby increased electron transport rate and PSII efficiency. However, to test this hypothesis further experiments are required. We also observed in independent experiments using hyperspectral imaging that application of okanin (**12**) to the shoots of *A. retroflexus* resulted in an altered photosynthetic pigment composition. In particular, there was a significant reduction of the spectral index PRI, which is sensitive to changes in carotenoid pigments such as xanthophylls, indicating a reduced light use efficiency of PSII^24^ compared with control. In parallel, the spectral index ARI increased in these experiments, indicating an increased stress level as these changes can be attributed to weak or senescing leaves^25^. A different formulation of the active compounds could result in generalized growth reduction over a longer time period of plant developmental stages and in a broader spreading of these molecules locally applied to a larger portion of the surrounding green tissues through long distance transport.

Previous studies have stressed the importance of the specific number and position of hydroxyl groups of chalcone derivatives for their efficacy^26^,^27^. Both okanin (**12**) and **10** are the only chalcones tested here with a 2′,3′,4′-trihydroxy substitution pattern in ring A. Together with the above structure-activity relationships, this suggests that this pattern is important for both *in vitro* inhibitory potency as well as *in planta* efficacy.

Our studies of the anti-microbial effects revealed that none of the chalcones tested here prevented growth of the four tested bacteria, except for **5**, which is the only chalcone modified at the 5′ position. Effects (increased duration of lag phase or reduced growth rate) were observed almost exclusively for the Gram-positives *B. subtilis* and *C. glutamicum*, which is in accordance with previous studies^28^,^29^. However, except for **5**, the concentrations of the chalcone derivatives influencing lag phase or growth rate of the bacteria were above the IC_50_ of C_4_ PEPC. Regarding potential pharmacological or toxicity effects of those chalcones that showed an influence on plants, we are only aware of two studies: Okanin (**12**) has been identified as a promising anticancer agent acting on human telomerase at an IC50 ~10-fold higher than for inhibition of C_4_ PEPC^26^; compound **10** has shown antiproliferative activity against human breast cancer cells at an IC50 ~5-fold higher than for inhibition of C_4_ PEPC^27^.

In conclusion, we propose that certain hydroxyl derivatives of the chalcone family, such as okanin (**12**) or **10**, which selectively inhibit C_4_ PEPC, can be applied as selective and environmentally sustainable herbicides against C_4_ weeds. Given the worldwide agronomic importance of C_3_ crops including rice, wheat, soybeans, fine grains, and legumes, a herbicide based on these polyhydroxy chalcones could substantially improve global crop production.

## Methods

### Chemicals

Chemical were from Sigma-Aldrich (St Louis, USA) if not stated otherwise. Okanin was from AApin Chemicals (Abingdon, UK). Robtein was from Synchem UG & Co. KG (Felsberg, Germany). The other compounds were from Ambinter c/o Greenpharma (Orléans, France).

### Cloning, protein expression and purification

*F. trinervia* and *F. pringlei* PEPC were cloned, expressed and purified as described previously^4^. Briefly, the full-length ppcA gene of PEPC from *F. trinervia* (EMBL-Bank X61304) and *F. pringlei* (EMBL-Bank Z48966) were cloned in pETEV16b (Novagene). The proteins were expressed in *Escherichia coli* strain BL21(DE3) (*F. trinervia*) or BL21-Gold(DE3) (*F. pringlei*) (Agilent Technologies) at 16 °C upon induction with 0.5 mM isopropyl-β-D-thiogalactoside. The proteins were purified by affinity chromatography with nickel column and changed to a final buffer containing 50 mM Tris/HCl pH 7.5, 150 mM NaCl, 10 mM MgCl_2_ using PD10 columns (GE Healthcare).

### PEPC coupled spectrophotometric assay

The IC_50_ values of small molecule compounds on *F. pringlei* and *F. trinervia* PEPCs were determined using PEPC coupled spectrophotometric assay^30^. The amount of NADH oxidized by NADH-malate dehydrogenase was measured by the decrease in absorbance at 340 nm in a Beckman DU-800 spectrophotometer, thus the value is in proportion to the corresponding substrate conversion of the enzyme. The reaction mixture included the PEPC enzymes (0.05 U), NADH (150 μM), malate dehydrogenase (2 U) and different concentration of compounds in the buffer containing 50 mM HEPES/KOH pH 7.5, 10 mM MgCl_2_, 10 mM KHCO_3_ in a final volume of 600 μl at 25 °C. The reactions were started by adding two folds of the K_m_ value of PEP for each enzyme. The data were analyzed using GraFit (Erithacus Software, UK).

### Isothermal titration calorimetry (ITC)

The binding affinities between small molecule compounds and PEPC enzymes were measured using a MicroCal iTC200 calorimeter (GE Healthcare). 18 injections (2 μl each) of 200 μM compound were injected into a sample cell containing 20 uM PEPC at 25 °C. The buffer of the compounds and the enzymes contained 50 mM Tris/HCl pH 7.5, 150 mM NaCl, 10 mM MgCl_2_. The data were analyzed with one-site-binding fitting using Origin software (MicroCal Inc.) to calculate the dissociation constant (K_d_) of the small molecule inhibitors.

### Docking with GLIDE

The protein and ligand structures were prepared as described in the Supplementary Information. We used the GLIDE module^31^ in extra-precision (XP) mode^32^,^33^ and default values for the grid generation. We centered the grid on the co-crystallized aspartate in the allosteric binding pocket of PEPC from *F. trinervia* (PDB ID 3ZGE) and *F. pringlei* (PDB ID 3ZGB). No restraints were used during the docking. Variants of the ligands with deprotonated *para* hydroxyl groups in ring A were docked, as done previously^34^.

### Minimization of ligand poses

The MAB force field^35^,^36^ as implemented in the program Moloc was used to minimize the docking poses in the binding pocket. For this, the protein atoms were defined as stationary, and only the ligand was allowed to move.

### Molecular dynamics simulations

MD simulations were carried out for **12** in the allosteric feedback inhibitor site of C_4_ PEPC (PDB ID 3ZGE) and **12** in C_3_ PEPC (PDB ID 3ZGB). The preparation of the ligands and the setup of the simulations were performed with programs from the Ambertools15 package^37^. After thermalization, three independent all-atom MD simulations of 200 ns length for each complex were performed with the AMBER14 suite using the GPU-accelerated pmemd program^37^,^38^. Root mean square deviations (RMSD) of the atomic coordinates were calculated with ptraj^39^ for the receptor with respect to the starting conformation, for residues of the binding site only, and for the ligand. In the latter case, only atoms of the protein were superimposed to also monitor movements of the ligand within the binding site. Further details are given in the Supplementary Information.

### AFMoC (Adaption of Fields for Molecular Comparison) analysis

A structure-based QSAR analysis was performed by the Adaption of Fields for Molecular Comparison (AFMoC) approach^13^,^40^,^41^. Further details are given in the Supplementary Information.

### *In planta* experiments

Seeds of *Amaranthus retroflexus* L. (Herbiseed, Twyford, UK) and *Brassica napus* L. cv. Mozart (Norddeutsche Pflanzenzucht Hans-Georg Lembke KG, Holtsee, Germany) were sown in vermiculite for germination. When cotyledons were fully expanded, seedlings were transplanted into plastic pots with a volume of 0.230 L filled with a substrate containing 50% bottom ash (0–4 mm), 30% peat and 20% pumice gravel (0–4 mm). Plants were watered every other day with 0.03% Hakaphos^®^ Grün solution (Compo Expert GmbH, Münster, Germany) by sub-irrigation. Plants were grown in a climate chamber at 22 °C/20 °C day/night temperature and a day/night length of 16 h/8 h. Relative humidity was adjusted to 70% and light intensity was 320 μmol m^−2 ^s^−1^ PAR (Photosynthetically Active Radiation) at plant level.

When seedlings reached the two-leaf growth stage, plant shoots were sprayed from the top with treatment solutions using spray bottles with a spraying angle of 35° (10 ml, neoLab Migge Laborbedarf-Vertriebs GmbH, Heidelberg, Germany). Treatment solutions were applied from a height of 8.5 cm above the pots. Each pot was sprayed once with a solution volume of 0.135 ml. Treatment solutions contained 2.4% (v/v) DMSO (dimethylsulfoxide, Duchefa Biochemie, Haarlem, the Netherlands), 0.1% (v/v) Tween^®^ and the respective compound at a concentration of 3 mM. The control treatment contained 2.4% (v/v) DMSO, and 0.1% (v/v) Tween20. Further details are given in the Supplementary Information.

CO_2_-response curves were obtained on *A. retroflexus* and *B. napus* using a LI-6400XT portable photosynthesis system equipped with a fluorescence head and a leaf chamber size of 2 cm^2^ (LI-COR, Lincoln, NE, USA) at a photon flux density of 1500 μmol m^−2 ^s^−1^ and at 25 °C leaf temperature^42^. Plants were grown at the research greenhouse of the IBG-2 Institute of Plant Sciences at Forschungszentrum Jülich, Germany (50°55′20″N 06°21′30″E), at a day/night length of 16 h/8 h, at an average day/night temperature of 23 °C/17 °C, relative humidity of 55%, and light intensity of 170 PAR. Plants were treated as described above at the three-leaf stage. Measurements were performed on the third leaf before treatment and one day after treatment. Each treatment was repeated four times. Information on statistical analysis is given in the Supplementary Information.

### Bacterial strains and cultivation conditions

The following bacteria were cultivated in the presence of *trans*-chalcone derived inhibitors: *Corynebacterium glutamicum* ATCC13032^43^, *Escherichia coli* K-12 MG1655^44^, *Pseudomonas putida* KT2440^45^, and *Bacillus subtilis* 168^46^. The growth of these bacteria was monitored in flower-well plates shaken at 1200 rpm as backscatter at 620 nm (gain 20) using a BioLector^®^ microscale cultivation system (m2p labs, Baesweiler, Germany). For further experimental details please refer to the Supplementary Information.

## Additional Information

**How to cite this article**: Nguyen, G. T. T. *et al*. Chalcone-based Selective Inhibitors of a C_4_ Plant Key Enzyme as Novel Potential Herbicides. *Sci. Rep.* **6**, 27333; doi: 10.1038/srep27333 (2016).

## Supplementary Material

Supplementary Information

## Figures and Tables

**Table 1:**
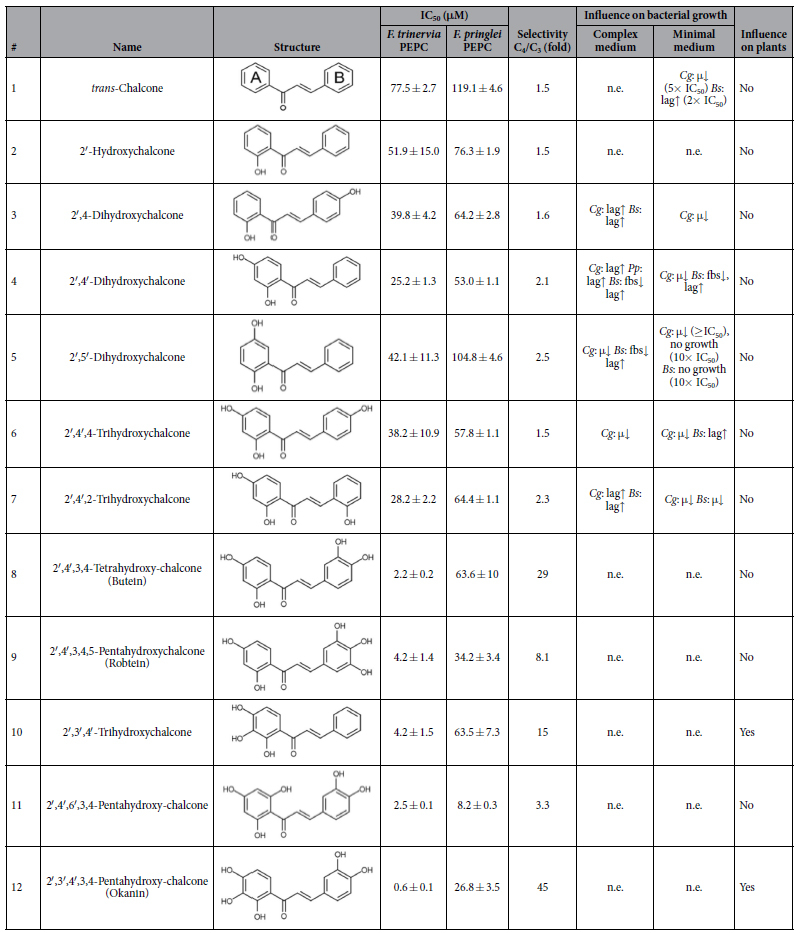
Inhibitory effects of *trans*-chalcone and its related compounds on *F. trinervia* and *F. pringlei* PEPC and their influences on the growth of three soil bacteria (*C. glutamicum* ATCC13032, *Cg*; *P. putida* KT2440, *Pp*; *B. subtilis* 168, *Bs*) and *E. coli* K-12 MG1655 (*Ec*).

IC_50_ determination of chalcones on the C_3_ and C_4_ PEPCs using activity assays. The selectivity of chalcones on the C_4_ PEPC over C_3_ isoform is also indicated in the Table. Error bars represent standard errors of at least two independent measurements. The strains were cultured in a microscale cultivation system both in complex and minimal media in the presence of 0.1×, 1×, and 10× the IC_50_ concentration determined for the *F. trinervia* PEP carboxylase. The Table indicates whether the final cell density (measured as backscatter at 620 nm) or the growth rate decreased (fbs↓ and μ↓, respectively), or the lag phase was extended (lag↑). All results refer to the highest chalcone concentration tested if not stated otherwise. Only effects leading to differences ≥10% compared to the untreated control cultures were included. Effects due to precipitation in the growth media occurring at 10× IC_50_ of *trans*-chalcone and 2′-Hydroxychalcone were not included to this Table. Abbreviations: n.d., not determined; n.e., no effect on growth.

**Table 2:** Influence of okanin on maximal PEP carboxylation rate (V_pmax_) of *A. retroflexus* estimated from leaf gas exchange measurements.

		Maximal PEP carboxylation rate (μmol m^−2 ^s^−1^)		*P*
		Before treatment	One day after treatment	
*A. retroflexus*	Untreated	31.2 (4.1)	29.3 (3.82)	0.740
	control_DMSO_	30.8 (3.57)	30.4 (3.37)	0.930
	Okanin	36.5 (3.96)	17.2 (2.43)	<0.001

Standard errors of estimates are given in brackets. *p*-values indicate significant differences at α = 0.05 between V_pmax_ values estimated from data obtained before treatment and one day after treatment. (n = 4).
