# Supplementary material for: Chalcone-based Selective Inhibitors of a C4 Plant Key Enzyme as Novel Potential Herbicides
Source: Sci Rep. 2016 Jun 6;6:27333. doi: 10.1038/srep27333 (PMC4893628; doi:10.1038/srep27333)
Supplement: Supplementary Information [file srep27333-s1.pdf]

Chalcone-based Selective Inhibitors of a C_4 Plant Key Enzyme as Novel Potential Herbicides

SUPPLEMENTARY INFORMATION

G.T.T. Nguyen[1], G. Erlenkamp[2], O. Jäck[3], A. Küberl[4], M. Bott[4], F. Fiorani[3], H. Gohlke[2], and G. Groth[1*]

[1]Biochemical Plant Physiology and [2]Pharmaceutical and Medicinal Chemistry, Heinrich Heine University Düsseldorf and Bioeconomy Science Center (BioSC)

[3]Institute of Bio- and Geosciences, IBG-2: Plant Sciences and [4]Institute of Bio- and Geosciences, IBG-1: Biotechnology, Forschungszentrum Jülich and Bioeconomy Science Center (BioSC)

* Correspondence and requests for materials should be addressed to G. G.:

Heinrich Heine University, Biochemical Plant Physiology, Universitätsstr. 1, 40225 Düsseldorf, Germany, Phone: +49-211-81-12822, Fax: +49-211-81-13569, Email: Georg.Groth@hhu.de

Supplemental Methods

Protein structure preparation

We retrieved the crystal structures of PEPC of the C_4 plant *F. trinervia* (PDB ID 3ZGE) and the C_3 plant *F. pringlei* (PDB ID 3ZGB)[1] from the Protein Data Bank[2]. Both structures were preprocessed with the Protein Preparation Wizard[3-7] of the Schrödinger suite. Bond orders were assigned, hydrogens were added, the H-bond network was optimized, and missing side chains were detected and added using Prime[8,9]. Finally, the systems were energy minimized using the OPLS 2005 force field resulting in a root mean square deviation (RMSD) of 0.3 Å with respect to the crystal structure[10-12]. Since the allosteric binding site is not affected by other monomers, we only used one monomer for further analysis[13,14].

Ligand preparation

The structures of the twelve ligands were sketched with ChemDraw 14[15]. 3D structures were generated with the LigPrep[16] module of the Schrödinger suite. We calculated the pKa values of all compounds with the Epik module[4,17,18]. For the *para* hydroxyl groups in ring A (see Table 1), a mean pKa value of 7.9 ± 0.8 was computed. This value is in line with experimental values reported for derivatives[19].

Molecular dynamics simulations

MD simulations were carried out for **12** inside the allosteric feedback inhibitor site of C_4 PEPC (PDB ID 3ZGE) and **11** inside of C_3 PEPC (PDB ID 3ZGB). The preparation of the ligands and the setup of the simulations were performed with programs from the Ambertools15 package[20]. Antechamber[21] was used to parameterize the compounds. Atomic charges were computed with

the AM1-BCC method[22,23]. The ff99SB force field[24] was used for the protein, and the General Amber Force Field (GAFF)[21] was used for the compounds. All co-crystallized components, such as water, 1,2-ethandiol, sulfate, and the inhibiting aspartate were removed from both used crystal structures prior to the following preparation of the complexes. LEaP was used to generate complex models from the ligand and receptor structures. After neutralizing the systems by adding 19 or 20 Na^+ ions according to the charge of the ligand, the systems were placed inside a truncated octahedral periodic box of TIP3P water[25] with a distance between the box edges and the closest atom of the complex of at least 10 Å. The size of the systems was ~92,000 atoms. Three independent MD simulations for each complex were performed with the AMBER14 suite using the GPU-accelerated pmemd program[20,26]. Initially, the solvated systems were energy minimized by 500 steps of steepest descent, followed by 500 steps of conjugate gradient minimization. The non-bonded cutoff was set to 8.0 Å, and the positions of the complex atoms were restrained with a harmonic potential using a force constant of 2.0 kcal mol^{-1} $Å^{-2}$. Bonds involving hydrogen atoms were constrained using the SHAKE algorithm[27]. After this step, the temperature was increased from 0 K to 300 K during 50 ps of NVT (canonical ensemble) MD simulations. The density of the solvent was adjusted by MD simulations of 50 ps length in the NPT (isothermal-isobaric) ensemble. The final equilibration step was performed for 500 ps during which the force constant for the harmonic restraints was set to zero. For all MD simulations, a time step of 2 fs was used. Production simulations were set up with a coupling time for the heat bath of 2.0 ps. The production runs achieved lengths of 200 ns.

AFMoC (Adaption of Fields for Molecular Comparison) analysis

One AFMoC model each was derived for the ligands within C_4 and C_3 PEPC (PDB IDs 3ZGE and 3ZGB), respectively. The ligand superimpositions were obtained by docking with GLIDE and subsequent minimization with the MAB force field as described above. DrugScore[28] was used to compute receptor-based potential fields[29] for four atom types present within the set of ligands (atom-type notation according to Sybyl[30]: C.2, C.ar, O.3,O.2). The grid box was set to a size such that all ligands were embedded with a margin of at least 4 Å, and the grid spacing was set to 1 Å. The half width of the Gaussian to compute interaction fields from the potential fields (see ref.[29] for more details) was set to 0.85 Å. Leave-one-out (LOO) analyses were performed. Aromatic carbons and hydroxyl oxygens of the ligands contribute most to the explanation of the variance in the binding affinities of the ligands (57% and ≥37 %, respectively). Due to the limited size of the data set further leave-multiple-out crossvalidations were not performed. Additionally, LOO-analyses after random scrambling of the biological data ("y-scrambling") were performed. They resulted in $q^2 < 0$ for both C_4 and C_3 PEPC, indicating that, with the original biological data, no chance correlation is given.

Experimental design and assessments of in planta experiments

We tested eleven compounds in a first experiment. In a follow-up experiment we tested selected compounds that showed growth inhibitory effects in the first experiment and one additional chalcone. Each treatment was repeated 12 times, except for oilseed rape in the second experiment (n=10). Pots were arranged in the growth chamber according to a fully randomized design. Initial growth of the second leaf of *A. retroflexus* plants was monitored by aquiring images of each plant from above from day zero to day two after treatment. Images were taken

from a fixed distance at 90° view angle using the camera Canon EOS 70D equipped with a Canon EF 14 mm/2.8 L II USM lens. Leaf area of single leaves was measured using the ImageJ software[31], by drawing the leaf contours using a drawing tablet (Cintiq 22HD, Wacom Europe GmbH, Krefeld, Germany). Conversion of pixel to cm was achieved by using a target of known size inside the image. Six days after the treatment, half of the plants were harvested. Leaf area was assessed using a leaf area meter (LI3100C Area Meter, LI-Cor, Lincoln, Nebraska, USA) and samples were oven-dried at 65°C for seven days to obtain dry weight. Thirteen days after the treatment, this procedure was repeated with the second half of plants.

Chlorophyll fluorescence measurements

For assessment of photosynthetic parameters, *A. retroflexus* plants were grown under the abovementioned conditions until the two-leaves growth stage. 6 mm leaf discs were cut out of the second leaf and immediately placed into 96-well-plates filled with 300 µL 0.125 % Hoagland's solution with 1 mM MES-KOH (MES-monohydrate, Merck, Darmstadt, Germany) buffered to a pH of 6.5. The treatment solution contained 1.6 % (v/v) DMSO, 0.1 % (v/v) Tween®20 and 2',3',4',3,4-Pentahydroxychalcone at a concentration of 2 mM. The control treatment contained 1.6 % (v/v) DMSO, 0.1 % (v/v) Tween®20. Each leaf disc was treated with 1.5 µL solution placed on top of the leaf discs. Plates were sealed with Parafilm and placed into a climate cabinet with 12h/12h day/night-length and a temperature of 22°C at a light intensity of 130 µmol m^{-2} s^{-1}. 96 hours after treatment, chlorophyll fluorescence measurements were performed on leaf discs using an Imaging PAM M-Series fluorescence camera (Heinz Walz GmbH, Effeltrich, Germany). Leaf discs were dark adapted for 30 minutes, minimum and maximum chlorophyll fluorescence were obtained and leaf discs were illuminated for 30 minutes

with a light intensity of 130 µmol m^{-2} s^{-1} PAR. Light curves were measured by stepwise PAR increments (0, 1, 21, 56, 111, 281, 396, 611, 801, 1251 µmol m^{-2} s^{-1} PAR). Leaf discs were illuminated at each intensity level for 30s before measuring fluorescence and maximum fluorescence by applying a saturation pulse of 2700 µmol m^{-2} s^{-1} PAR[32].

Hyperspectral imaging of A. retroflexus plants

Images were taken with the hyperspectral line-scanner Pica XC (Resonon, MT; USA) equipped with Schneider Xenoplan lens with 23 mm focal length (Jos. Schneider Optische Werke GmbH, Bad Kreuznach, Germany). The line-scanner is integrated in a system for automatic transport and positioning of sensors in a greenhouse[33] and is placed inside a closed box to prevent light from outside disturbing the measurements. The box is equipped with two 400 Watt halogen lamps providing measurement light. Images were taken with an integration time of 50 ms, a framerate of 20 s^{-1}, a scanning speed of 4 mm s^{-1} and a spectral resolution of 4.37 nm. Plants were cultivated as in the other experiments, except that temperature and light conditions differed. Temperature conditions in the greenhouse were 19°C/17°C at a day/night length of 16h/8h and a light intensity of approximately 85 µE PAR m^{-2} s^{-2}. Relative humidity was approximately 60 %. Plants were treated in the three-leaves stage and treatment solutions were prepared as mentioned before. Each treatment had six replicates. Before recording hyperspectral images, dark current was measured by covering the objective lens. Digital numbers of recorded hyperspectral images were transformed into reflectance by setting them in relation to a calibrated reflectance target (Spectralon, SpehereOptics, Uhldingen, Germany) that was placed inside the image, after the dark current spectrum was subtracted from both reflectance target and sample spectra. Mean spectra of the 3rd leaf of *A. retroflexus* were extracted as well as the corresponding spectra of the

reflection target. Photochemical reflection index (PRI) was calculated according to Gamon et al.[34] and anthocyanin reflectance index (ARI) was calculated according to Gitelson et al.[35]. For false-color images, data cubes were segmented by using a threshold based on the normalized difference vegetation index (NDVI) in combination with a NIR-threshold at 830 nm to separate plant pixels from background. ARI and PRI were calculated separately for each pixel. Image processing was performed using R Software for Statistical Computing version 3.1.1[36]. The package 'hyperSpec'[37] was used to read data files in bil-format.

Statistical Analysis of in planta experiments

All analysis were performed using R Software for Statistical Computing version 3.1.1[36]. Leaf area and biomass data were first analysed separately for each experiment, because treatments differed. To test whether effects of okanin (**12)** and (**10**) on *A. retroflexus* were consistent over both experiments, the combined data of both experiments were analyzed including 'experiment' as a categorical two-level factor Analyses of variance were performed with $\alpha=0.05$. Shapiro-Wilk tests were performed to test for normal distribution of residuals, and homogeneity of residuals was checked visually. Comparisons of means were conducted using Tukey's honest significance test using the package 'agricolae'[38]. Analyses of variances were performed on spectral indices ($\alpha=0.05$). Fischer's LSD was calculated to compare treatment means using the package 'agricolae'[38]. Analysis of leaf growth data was performed using the R package 'nlme'[39]. A two-parametric exponential growth model was fitted to the data with compound symmetry structure including random effects for each parameter accounting for between-plant variability. We run the model including treatment effects as covariate for both parameters. However, covariate effects were not significant for the intercept parameter, whereas it was for the rate

parameter. Therefore we included the treatment effect only for the rate parameter. Gas exchange measurements were used to estimate maximal PEP carboxylation rate (V_{pmax}) of *A. retroflexus* according to the model of von Caemmerer[40] at intercellular CO_2 partial pressure smaller 70 µbar. A full model, with V_{pmax} varying for measurement dates, and a reduced model, with one common V_{pmax} for measurement dates, was fitted separately for each treatment and compared via F-test at $\alpha=0.05$. Mitochondrial respiration losses were not accounted for in the final models, because they occurred to have no significant effects in model-fitting. For data of *B. napus* we fitted the Farquhar-von Caemmerer-Berry model[41]. This model estimates maximal rubisco carboxylation rate (V_{cmax}), maximal electron transport rate (J_{max}) and mitochondrial respiration (R_d). The full model was fitted with separate parameters for measurement dates. The model was stepwise reduced to common parameters for measurement dates starting with the least significant R_d, followed by J_{max} and V_{cmax}. Reduced models were compared to the full model via an F-test at $\alpha=0.05$. Constants were taken from Sharkey et al.[42].

Bacterial cultivation conditions

C. glutamicum and *P. putida* were cultivated at 30°C, *E. coli* and *B. subtilis* at 37°C. All strains were cultivated in BHI complex medium (Difco Laboratories, Detroit, USA) supplemented with 2% (w/v) glucose and in appropriate minimal media containing 2% (w/v) glucose as carbon and energy source. The minimal media used were CGXII medium[43] for *C. glutamicum*, M9 medium[44] for *E. coli* and *P. putida*, and TS (Spizizen)[45] medium for *B. subtilis*. Deviating from the standard composition, TS medium was supplemented with M9 trace element solution[44] and 20 mg/L L-tryptophan, whereas no yeast extract was added. All strains were inoculated in three biological replicates in 5 ml BHI medium containing 2% glucose and cultivated for 6 hours at 170 rpm. Then, 285 µl of the pre-cultures were washed and inoculated into 20 ml of complex

medium or the appropriate minimal medium and shaken at 140 rpm overnight. For inoculation of the main culture, cells equivalent to an optical density at 600 nm of 0.5 (*C. glutamicum*, *E. coli*, and *P. putida*) or 0.25 (*B. subtilis*) in 800 µL medium were washed and resuspended in fresh media. Inhibitor substances were dissolved in DMSO and added to the media to a final concentration of the IC_{50} of the *F. trinervia* PEPC and 10-fold higher and lower concentrations. Since the least polar *trans*-chalcone and 2'-hydroxychalcone precipitated in the growth media at the highest concentrations, the maximal concentration of these compounds was reduced to 400 µM and 100 µM, respectively. The final backscatter at 620 nm, the growth rate (h^{-1}), and the duration of the lag phase of all cultures were determined in a BioLector microscale cultivation system in comparison to negative controls supplemented with the solvent DMSO. A longer lag phase was only assumed if the growth rates were comparable.

Supplemental Results

AFMoC (Adaption of Fields for Molecular Comparison) analysis

To substantiate the qualitative structure-activity relationship (SAR) for the chalcone compounds described in the main text, a quantitative SAR model using the protein-based AFMoC analysis was established[29].

For the ligand superimposition in C_4, a significant and good AFMoC model with a q^2 value of 0.63 for a leave-one-out cross-validation were obtained (Table S1 and Fig. S4a). Due to the limited range of IC50 values of the chalcones binding to C_3 PEPC (Table 1), no AFMoC model can be derived for this case[46]. STDEV*COEFF contour plots for hydroxyl oxygen elucidate regions where the presence of hydroxyl oxygens will enhance or reduce binding (Fig. S4b, c). Prominent contours indicating enhanced binding are visible in the vicinity of R641, K829, and N964 as well as close to L680 and R687. These results corroborate the importance of hydroxyl groups 2', 3', 4' in ring A and 3, 4 in ring B for okanin's (**12**) high affinity. In turn, the absence of hydroxyl groups at positions 3' and 4' of ring A and positions 3 and 4 of ring B in compound **5** explains its low affinity in both PEPC variants. Finally, a contour indicating reduced binding points towards G884, confirming that hydroxyl groups at positions 5' (in **5**) or 6' (in **11**) of ring A hamper binding to C_4 PEPC (Table 1).

Supplemental References

1 Paulus, J. K., Niehus, C. & Groth, G. Evolution of C4 phosphoenolpyruvate carboxylase: enhanced feedback inhibitor tolerance is determined by a single residue. *Mol Plant* **6**, 1996-1999 (2013).

2 Berman, H. M. *et al.* The Protein Data Bank. *Nucleic Acids Research* **28**, 235-242 (2000).

3 Schrödinger Suite 2014-1, Protein Preparation Wizard (Schrödinger, LLc, New York, 2014).

4 Schrödinger Suite 2014-1, Epik v. 2.7 (Schrödiner, LLC, New York, NY, 2013).

5 Schrödinger Suite 2014-1, Impact v. 6.2 (Schrödinger, LLC, New York, NY, 2014).

6 Schrödinger Suite 2014-1, Prime v. 2.5 (Schrödinger, LLC, New York, NY, 2014).

7 Sastry, G. M., Adzhigirey, M., Day, T., Annabhimoju, R. & Sherman, W. Protein and ligand preparation: parameters, protocols, and influence on virtual screening enrichments. *Journal of computer-aided molecular design* **27**, 221-234 (2013).

8 Jacobson, M. P., Friesner, R. A., Xiang, Z. & Honig, B. On the Role of the Crystal Environment in Determining Protein Side-chain Conformations. *Journal of Molecular Biology* **320**, 597-608 (2002).

9 Jacobson, M. P. *et al.* A hierarchical approach to all-atom protein loop prediction. *Proteins* **55**, 351-367 (2004).

10 Banks, J. L. *et al.* Integrated Modeling Program, Applied Chemical Theory (IMPACT). *J. Comput. Chem.* **26**, 1752-1780 (2005).

11 Jorgensen, W. L. & Tirado-Rives, J. The OPLS Potential Functions for Proteins. Energy Minimizations for Crystals of Cyclic Peptides and Crambin. *JACS* **110**, 10 (1988).

12 Gilson, M. K., Given, J. A., Bush, B. L. & McCammon, J. A. The Statistical-Thermodynamic Basis for Computation of Binding Affinities: A Critical Review. *Biophys. J.* **72**, 1047-1069 (1997).

13 Matsumura, H. *et al.* Crystal Structures of C4 Form Maize and Quaternary Complex of E. coli Phosphoenolpyruvate Carboxylases. *Structure* **10**, 1721-1730 (2002).

14 Muramatsu, M., Suzuki, R., Yamazaki, T. & Miyao, M. Comparison of plant-type phosphoenolpyruvate carboxylases from rice: identification of two plant-specific regulatory regions of the allosteric enzyme. *Plant Cell Physiol* **56**, 468-480 (2015).

15 ChemDraw v. 14.0 (PerkinElmer, 2014).

16 Schrödinger Suite 2014-1, LigPrep (Schrödinger, LLC, New York, NY, 2014).

17 Greenwood, J., Calkins, D., Sullivan, A. & Shelley, J. Towards the comprehensive, rapid, and accurate prediction of the favorable tautomeric states of drug-like molecules in aqueous solution. *Journal of computer-aided molecular design* **24**, 591-604 (2010).

18 Shelley, J. C. *et al.* Epik: a software program for pK(a) prediction and protonation state generation for drug-like molecules. *Journal of computer-aided molecular design* **21**, 681-691 (2007).

19 Rastelli, G., Antolini, L., Benvenuti, S. & Costantino, L. Structural bases for the inhibition of aldose reductase by phenolic compounds. *Bioorganic & Medicinal Chemistry* **8**, 1151-1158 (2000).

20 Amber14 (University of California, San Francisco, 2015).

21 Wang, J., Wolf, M., Caldwell, J. W., Kollman, P. A. & Case, D. A. Development and Testing of a General Amber Force Field. *Journal of computational chemistry* **25**, 17 (2004).
22 Jakalian, A., Bush, B. L., Jack, D. B. & Bayly, C. I. Fast, Efficient, Generation of High-Quality Atomic Charges. AM1-BCC Model: I. Method. *Journal of computational chemistry* **21**, 15 (2000).
23 Jakalian, A., Jack, D. B. & Bayly, C. I. Fast, efficient generation of high-quality atomic charges. AM1-BCC model: II. Parameterization and validation. *J. Comput. Chem.* **23**, 1623-1641 (2002).
24 Wang, J., Cieplak, P. & Kollman, P. A. How Well Does a Restrained Electrostatic Potential (RESP) Model Perform in Calculating Conformational Energies of Organic and Biologicel Moleculed. *Journal of computational chemistry* **21**, 26 (2000).
25 Jorgensen, W. L., Chandrasekhar, J., Madura, J. D., Impey, R. W. & Klein, M. L. Comparison of simple potential functions for simulating liquid water. *The Journal of Chemical Physics* **79**, 926 (1983).
26 Salomon-Ferrer, R., Götz, A. W., Poole, D., Le Grand, S. & Walker, R. C. Routine Microsecond Molecular Dynamics Simulations with AMBER on GPUs. 2. Explicit Solvent Particle Mesh Ewald. *Journal of chemical theory and computation* **9**, 3878-3888 (2013).
27 Miyamoto, S. & Kollman, P. A. SETTLE: An Analytical Version of the SHAKE and RATTLE Algorithm for Rigid Water Models. *Journal of computational chemistry* **13**, 10 (1992).
28 Gohlke, H., Hendlich, M. & Klebe, G. Knowledge-based Scoring Function to Predict Protein-Ligand Interactions. *J. Mol. Biol* **295**, 20 (2000).
29 Gohlke, H. & Klebe, G. DrugScore Meets CoMFA: Adaption of Fields for Molecular Comparison (AFMoC) or How to Tailor Knowledge-BAsed pair-Potentials to a Particular Protein. *Journal of medicinal chemistry* **45**, 17 (2002).
30 SYBYL-X v. 1.2 (Tripos International, 1699 South Hanley Rd., St. Louis, Missouri, 63144 USA, 2010).
31 Schneider, C. A., Rasband, W. S. & Eliceiri, K. W. NIH Image to ImageJ: 25 years of image analysis. *Nat Meth* **9**, 671-675 (2012).
32 Maxwell, K. & Johnson, G. N. Chlorophyll fluorescence—a practical guide. *Journal of Experimental Botany* **51**, 659-668 (2000).
33 Fanourakis, D. *et al.* Rapid determination of leaf area and plant height by using light curtain arrays in four species with contrasting shoot architecture. *Plant Methods* **10**, 9-9 (2014).
34 Gamon, J. A., Serrano, L. & Surfus, J. S. The photochemical reflectance index: an optical indicator of photosynthetic radiation use efficiency across species, functional types, and nutrient levels. *Oecologia* **112**, 492-501 (1997).
35 Gitelson, A. A., Merzlyak, M. N. & Chivkunova, O. B. Optical Properties and Nondestructive Estimation of Anthocyanin Content in Plant Leaves¶. *Photochemistry and Photobiology* **74**, 38-45 (2001).
36 Team, R. C. R: A language and environment for statistical computing. **R Foundation for Statistical Computing, Vienna, Austria** (2014).
37 Beleites, C. & Sergo, V. hyperSpec: a package to handle hyperspectral data sets in R. **R package version 0.98-20150304** (2015).

38 Mendiburu, F. d. agricolae: Statistical Procedures for Agricultural Research. **R package version 1.2-1** (2014).
39 Pinheiro, J., Bates, D., DebRoy, S., Sarkar, D. & Team, R. C. nlme: Linear and Nonlinear Mixed Effects Models. **R package version 3.1-120** (2015).
40 von Caemmerer, S. *Biochemical models of Leaf Photosynthesis. Techniques in Plant Sciences No 2.*, 96 (Collingwood: CSIRO Publishing, 2000).
41 Farquhar, G. D., von Caemmerer, S. & Berry, J. A. A biochemical model of photosynthetic CO2 assimilation in leaves of C3 species. *Planta* **149**, 78-90 (1980).
42 Sharkey, T. D., Bernacchi, C. J., Farquhar, G. D. & Singsaas, E. L. Fitting photosynthetic carbon dioxide response curves for C3 leaves. *Plant, Cell & Environment* **30**, 1035-1040 (2007).
43 Frunzke, J., Engels, V., Hasenbein, S., Gätgens, C. & Bott, M. Co-ordinated regulation of gluconate catabolism and glucose uptake in *Corynebacterium glutamicum* by two functionally equivalent transcriptional regulators, GntR1 and GntR2. *Molecular microbiology* **67**, 305-322 (2008).
44 Lee, D. H. & Palsson, B. O. Adaptive Evolution of *Escherichia coli* K-12 MG1655 during Growth on a Nonnative Carbon Source, L-1,2-Propanediol. *Applied and environmental microbiology* **76**, 4158-4168 (2010).
45 Hauser, P. M. & Karamata, D. A rapid and simple method for *Bacillus subtilis* transformation on solid media. *Microbiology* **140**, 1613-1617 (1994).
46 Thibaut, U. *et al.* in *3D QSAR in Drug Design. Theory, Methods and Applications* (ed H. Kubinyi) 711-716 (ESCOM, 1993).

Supplemental Figures

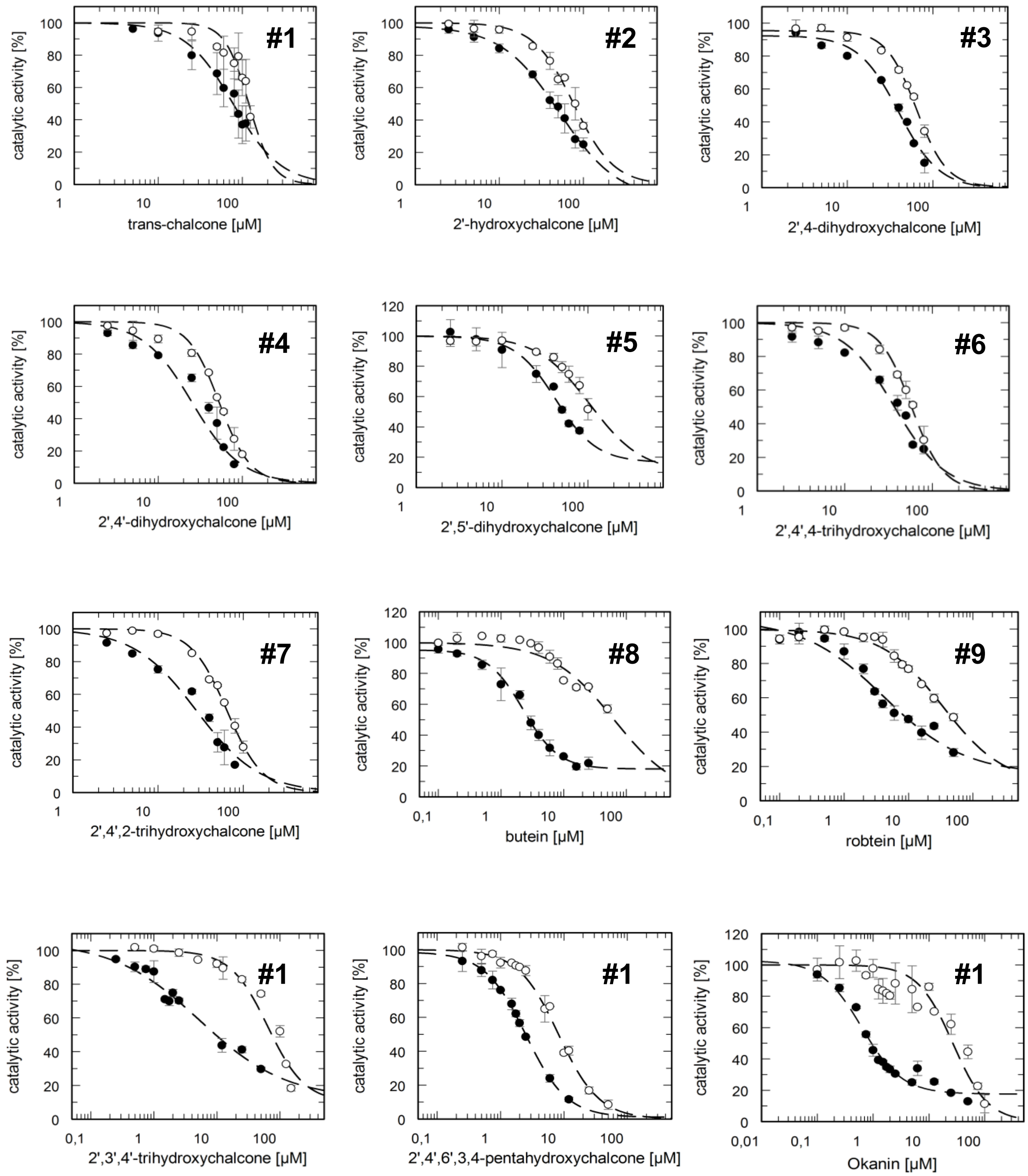

Figure S1: IC_{50} determination of 12 chalcones on PEPCs from *F. trinervia* and *F. pringlei* using activity assay.

Inhibition curves are fitted and the IC50 values are calculated for the C_4 (●) and C_3 (○) PEPC. Okanin (**12**) with the best potency and selectivity on C_4 over C_3 has the largest shift between two fitting curves. Error bars represent standard errors of two independent measurements.

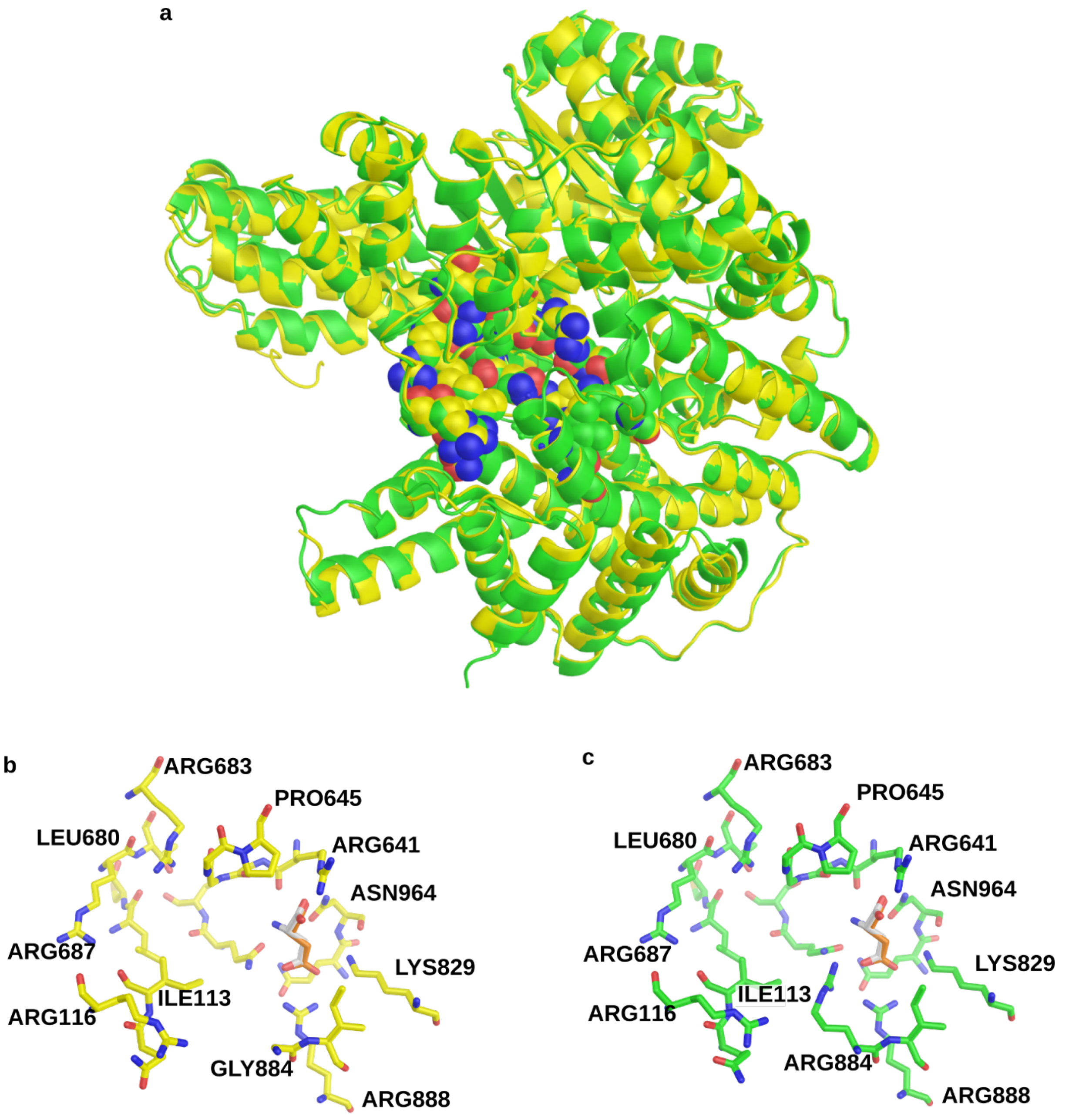

Figure S2: Redocking of Asp in C_4 and C_3 PEPC.

a, Overlay of C_4 (yellow) and C_3 (green) PEPC. Residues of the feedback inhibitor binding site are depicted as spheres. **b, c,** Redocking of aspartate in C_4 (b) and C_3 (c) PEPC. The co-crystallized aspartate is shown in grey, and the lowest energy docking pose is shown in orange.

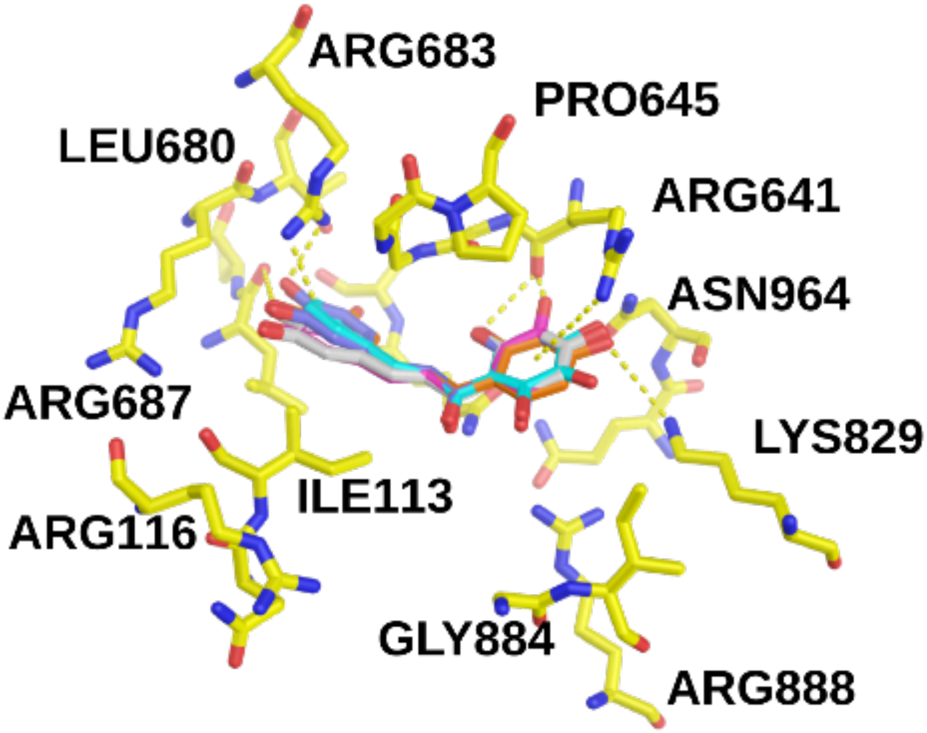

Figure S3: Common binding motif of tested chalcones and hydrogen bond network.

Overlay of binding poses of **8** (grey), **9** (orange), **10** (cyan), **11** (blue), and **12** (magenta) obtained by docking with GLIDE into C_4 PEPC. Dashed lines depict hydrogen bonds and cation-π-interactions between the ligands and the protein. The roman numbers label the hydrogen bonds as mentioned in Figure 1d and e.

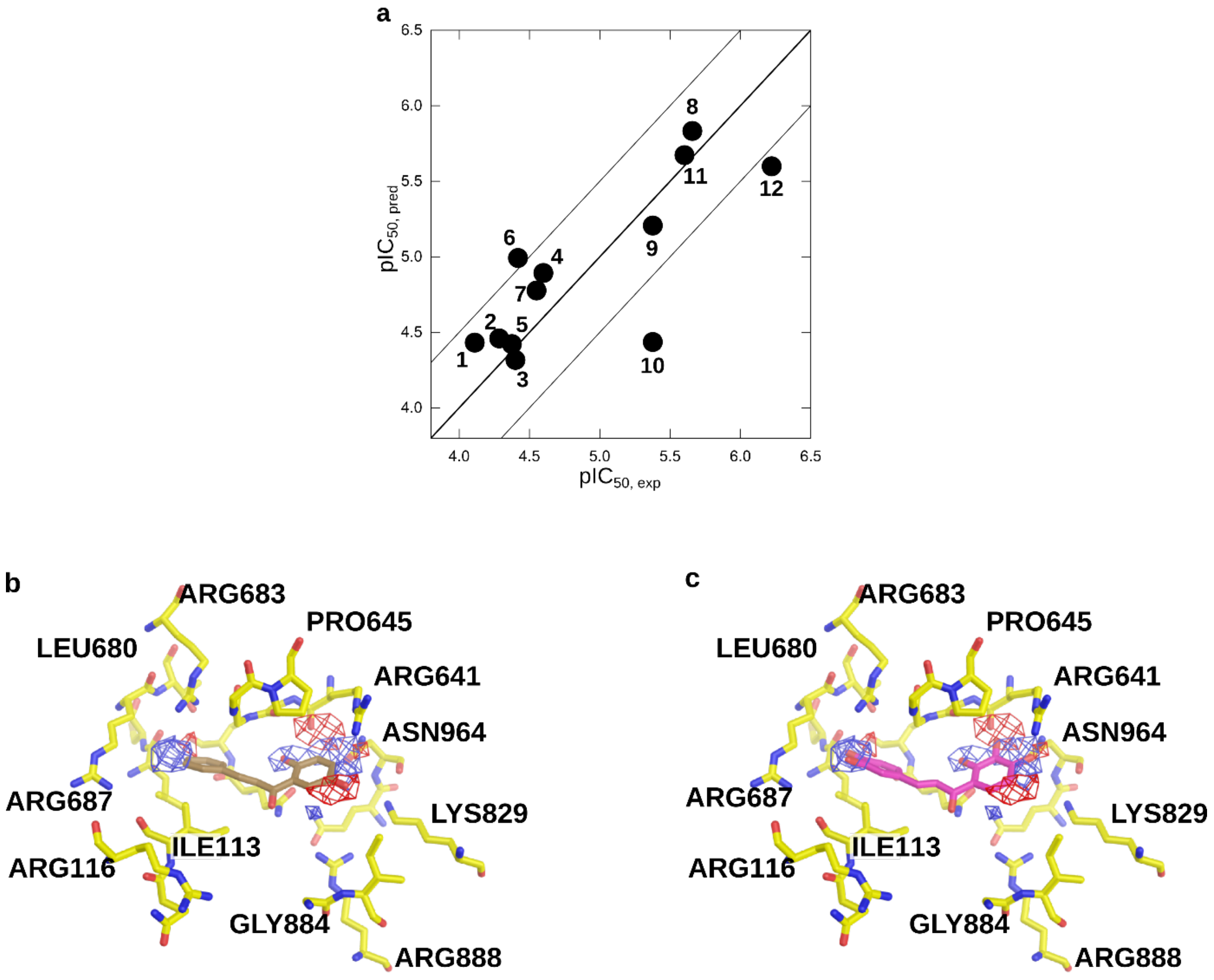

Figure S4: Results of the AFMoC analyses for chalcone ligands in C_4.

a, pIC_{50} values predicted by leave-one-out analysis versus experimentally determined ones for 12 chalcone ligands (see Table 1 in the main text). In addition to the line of ideal correlation, thin lines are given to indicate deviations of half a logarithmic unit from ideal prediction [24]. **b, c,** AFMoC STDEV*COEFF contour plots elucidating regions in the binding pocket of PEPC where the presence of hydroxyl oxygens will enhance (blue mesh) or reduce (red mesh) binding. Contour levels are -0.0075 and 0.02, respectively. Ligands 5 (b, brown; pIC_{50} = 4.357 in C_4 PEPC) and 12 (c, magenta; pIC_{50} = 6.22 in C_4 PEPC) are shown for comparison.

a

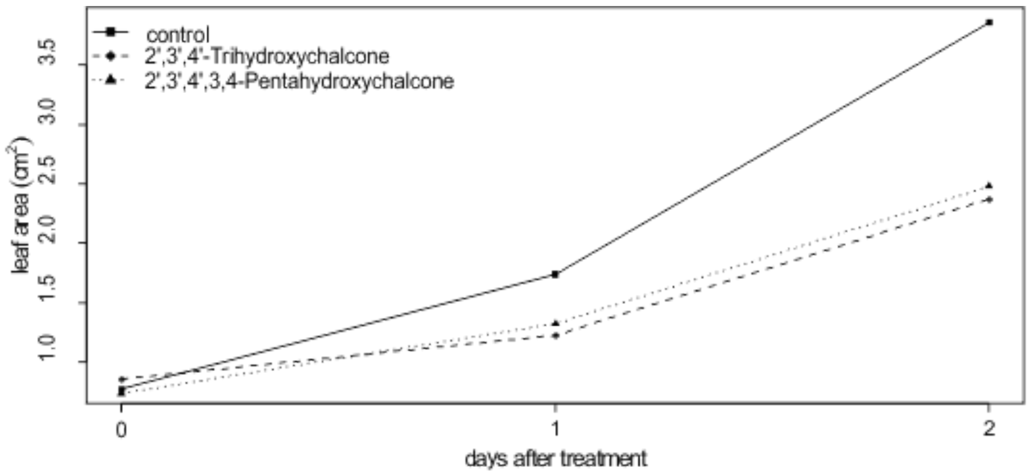

b

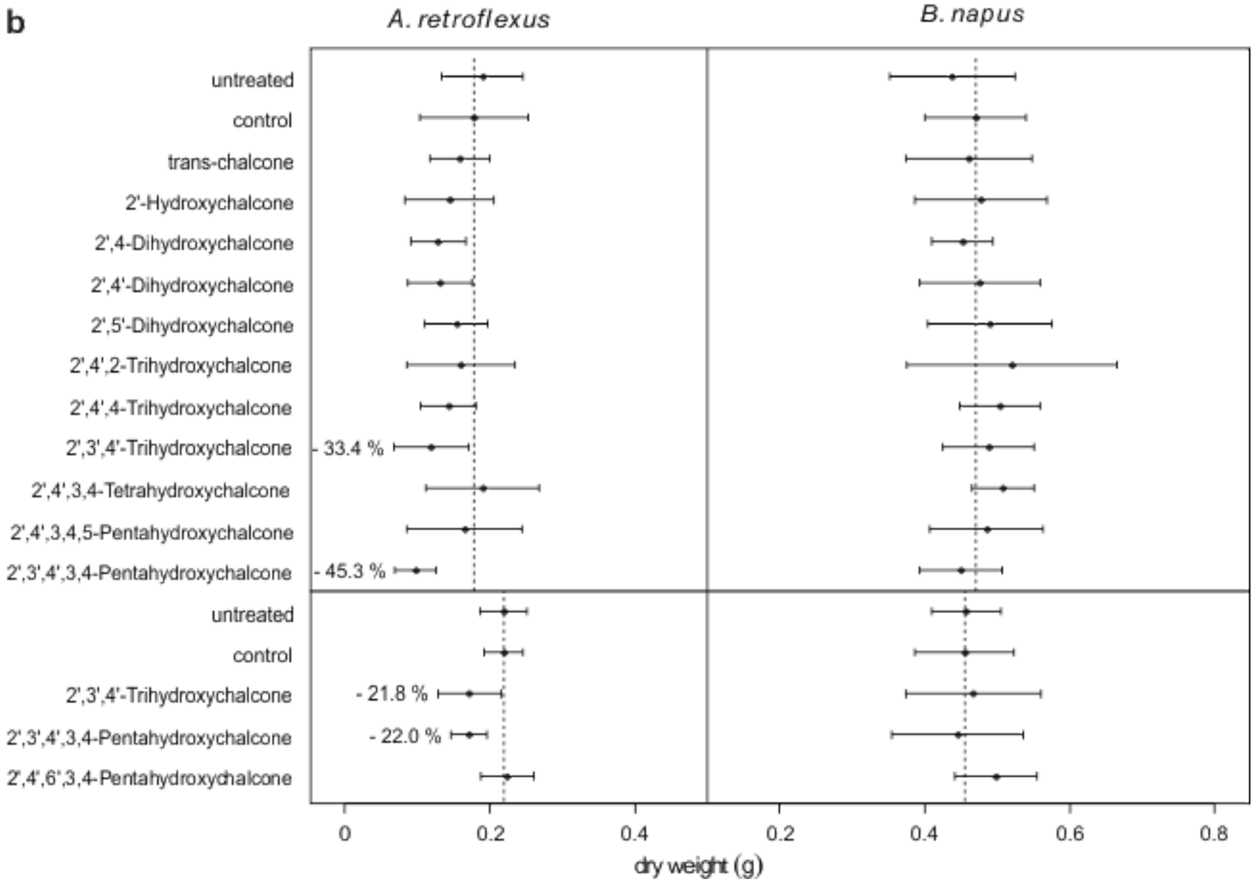

Figure S5: Influence of selected chalcones on leaf growth of *A. retroflexus* and biomass formation of *A. retroflexus* and *B. napus*

a, Growth of the 2nd leaf of *A. retroflexus* displayed as leaf area (cm²) at 0, 1 and 2 days after treatment with 3 mM 2',3',4'-Trihydroxychalcone (**10**) and 2',3',4',3,4-Pentahydroxychalcone (**12**) in comparison to the control treatment. Rate of growth was significantly reduced for the two displayed chalcones. **b**, Dry weight of *A.retroflexus* and *B.napus* six days after treatment.

Results of experiment 1 (top) and experiment 2 (bottom) are displayed. 2',3',4'-Trihydroxychalcone (**10**) and 2',3',4',3,4-Pentahydroxychalcone (**12**) significantly reduced *A. retroflexus* dry weight, and the effect was not significantly different between the two experiments. None of the compounds had an effect on dry weight of *B. napus*. Bars display standard deviations (n=6; *B. napus* experiment 2: n=5). Dashed lines represent the average of the corresponding control treatments.

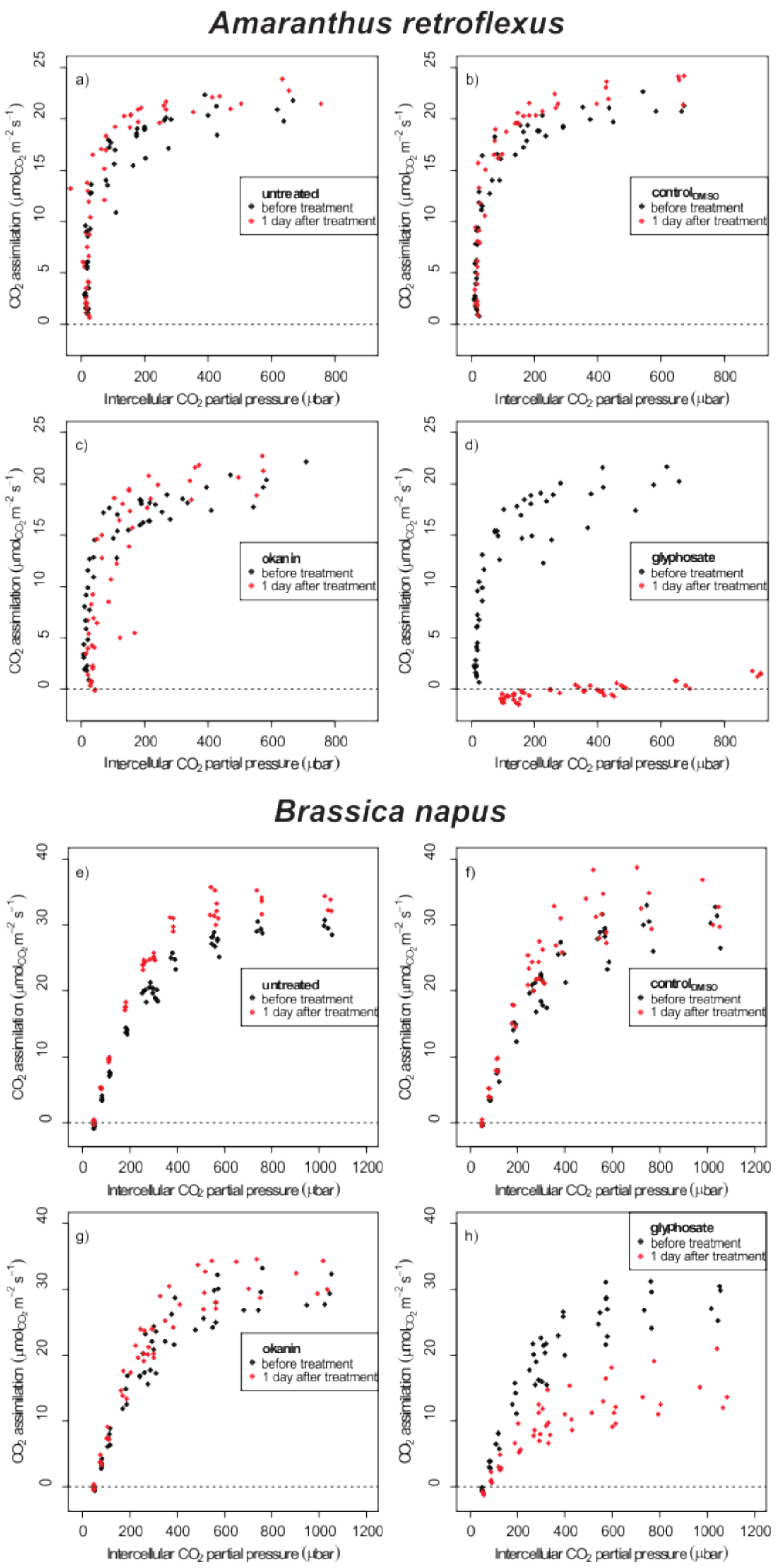
Amaranthus retroflexus
a)
untreated
before treatment
1 day after treatment
b)
control$_{DMSO}$
before treatment
1 day after treatment
c)
okanin
before treatment
1 day after treatment
d)
glyphosate
before treatment
1 day after treatment
CO_2 assimilation (μmol_{CO_2} m^{-2} s^{-1})
Intercellular CO_2 partial pressure (μbar)
Brassica napus
e)
untreated
before treatment
1 day after treatment
f)
control$_{DMSO}$
before treatment
1 day after treatment
g)
okanin
before treatment
1 day after treatment
h)
glyphosate
before treatment
1 day after treatment
CO_2 assimilation (μmol_{CO_2} m^{-2} s^{-1})
Intercellular CO_2 partial pressure (μbar)

Figure S6: Influence of okanin on CO_2 assimilation of *A. retroflexus* (a-d) and *B. napus* (e-h) before treatment and one day after treatment.

a, e, CO_2 assimilation of untreated plants. **b, f,** CO_2 assimilation of control (DMSO) plants. **c, g**, CO_2 assimilation of plants treated with okanin. **d, h,** CO_2 assimilation of plants treated with glyphosate**.**

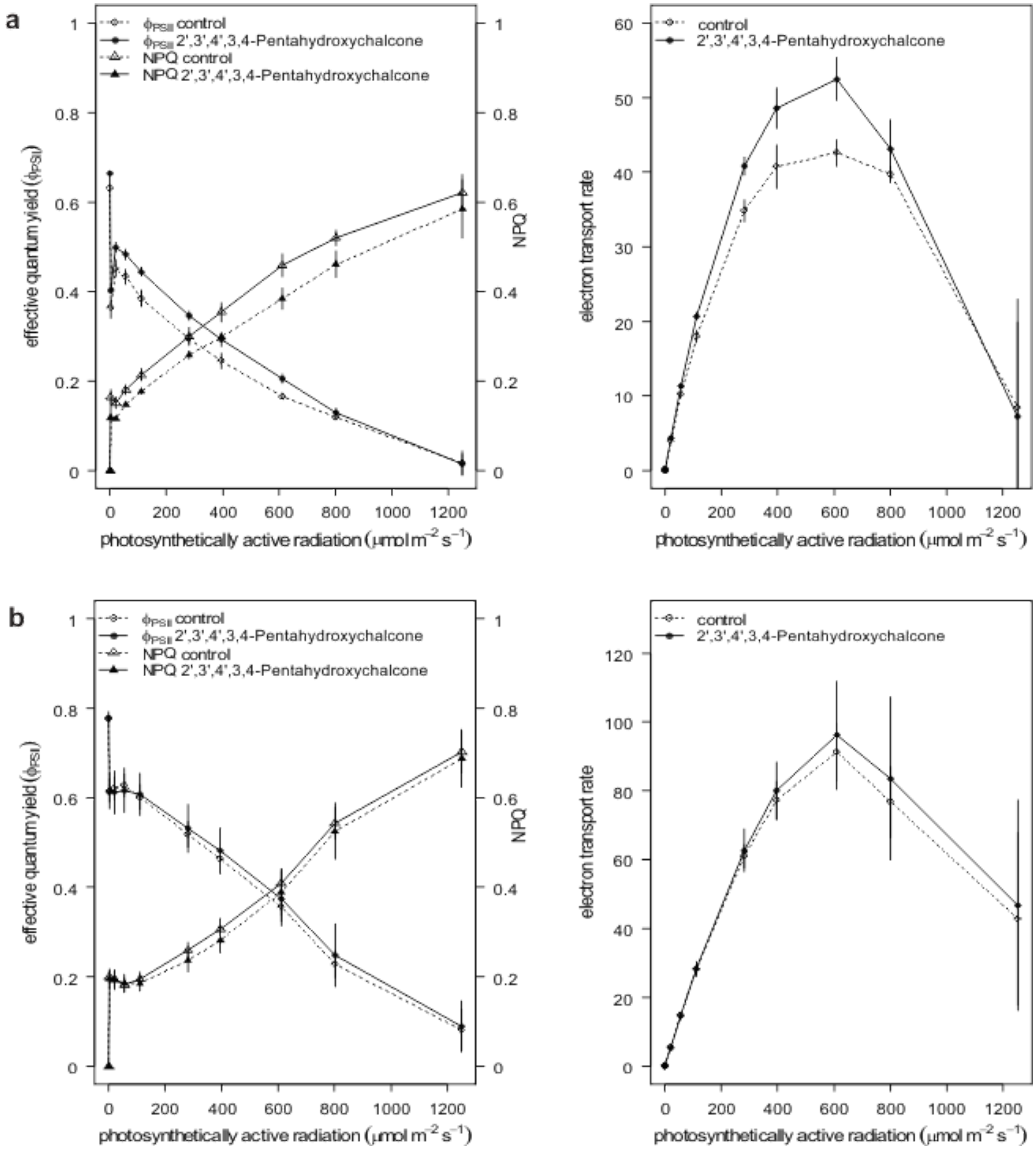

Figure S7: Influence of selected chalcones on photosynthetic parameters of *B. napus* and *A. retroflexus*

a, Influence of 2 mM 2',3',4',3,4-Pentahydroxychalcone on photosynthetic parameters of the 2nd leaf of *A. retroflexus* measured as rapid light curve in leaf disc assays. Effective quantum yield of photosystem II (Φ_{PSII}) was increased in comparison to the control treatment, whereas PSII non-photochemical quenching (NPQ) was reduced (left). Electron transport rate was increased

following treatment with 2 mM 2',3',4',3,4-Pentahydroxychalcone. Bars display standard deviation (n=4). **b**, Influence of 2mM 2',3',4',3,4-Pentahydroxychalcone on photosynthetic parameters of the 2nd leaf of A. *retroflexus* measured as rapid light curve in leaf disc assays. There was no effect on photosynthesic parameters of *B. napus*. Bars display standard deviation (n=4).

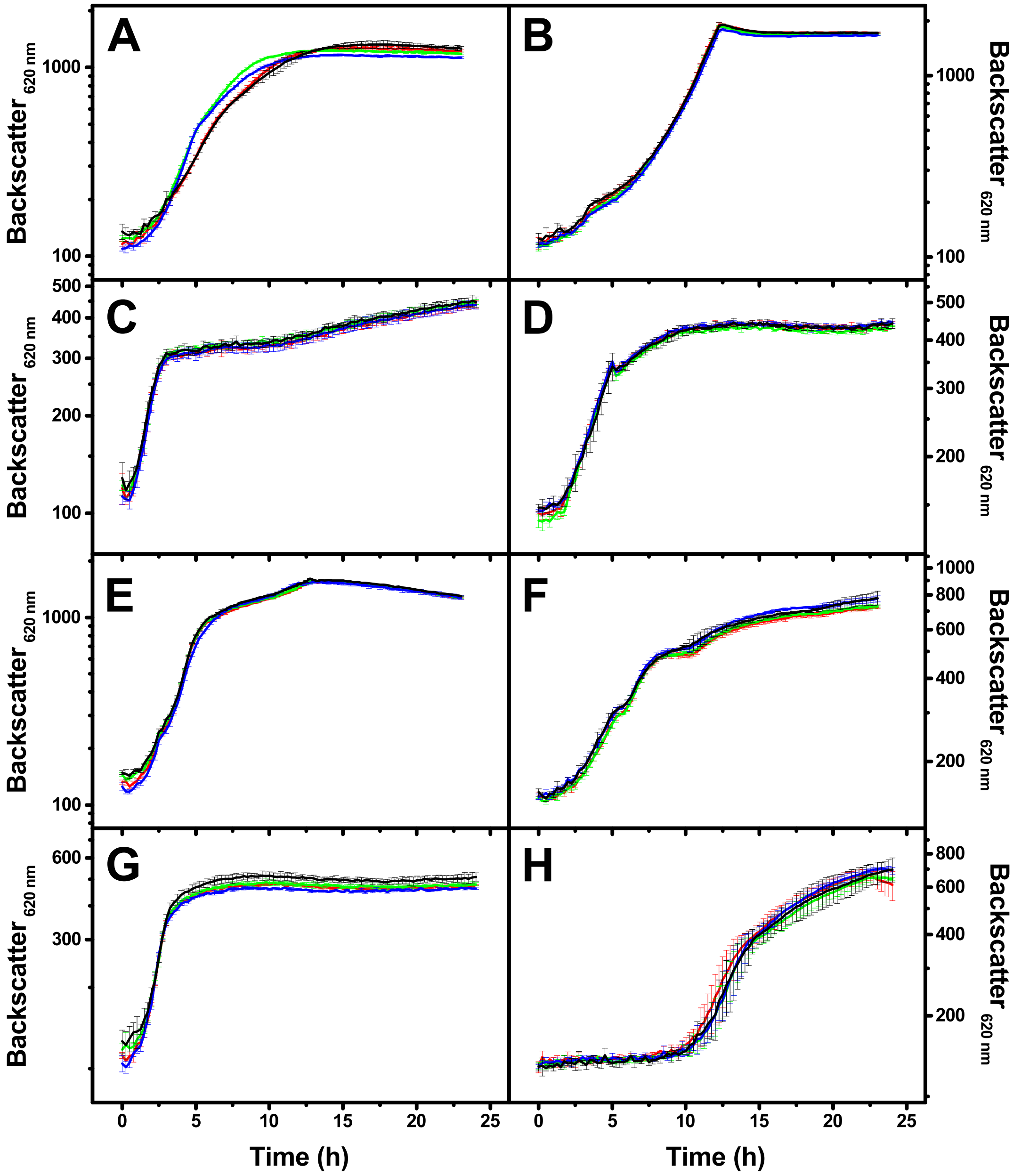

Figure S8: Cultivation of bacteria in the presence of okanin.

Cultivation of *C. glutamicum* ATCC 13032 (A, B), *E. coli* K-12 MG1655 (C, D), *P. putida* KT2440 (E, F), and *B. subtilis* 168 (G, H) in the presence of 0 µM (black), 0.1 µM (red), 1 µM (green), and 10 µM (blue) okanin (2',3',4',3,4-Pentahydroxychalcone). The cells were cultivated in complex media (BHI medium; A, C, E, G) or minimal media (CGXII, B; M9, D, F; Spizizen, H) containing 2% (*w*/*v*) glucose. The cell density was monitored in the Biolector system measuring the backscatter at 620 nm (gain 20).

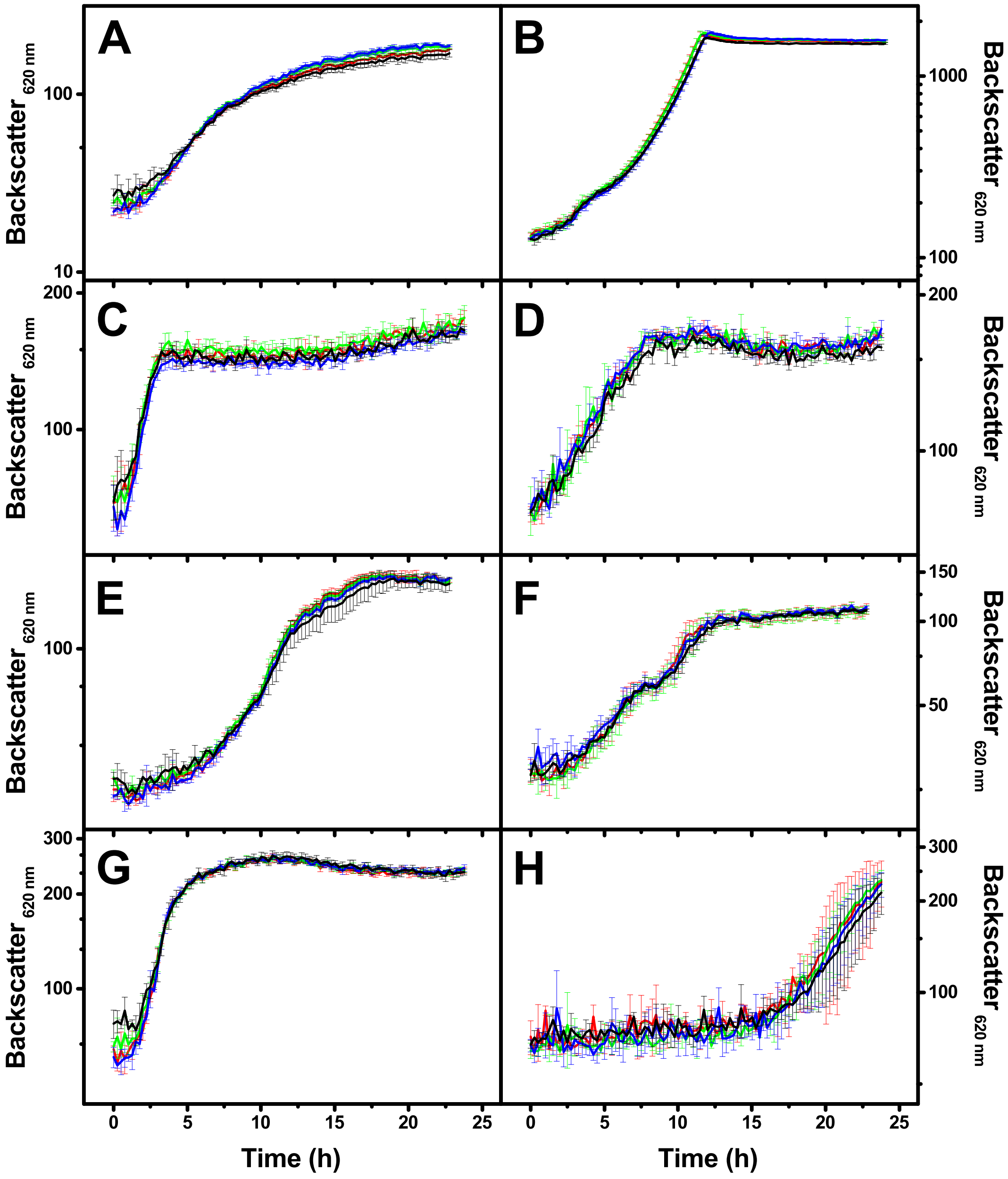

Figure S9: Cultivation of bacteria in the presence of chalcone 10.

Cultivation of *C. glutamicum* ATCC 13032 (A, B), *E. coli* K-12 MG1655 (C, D), *P. putida* KT2440 (E, F), and *B. subtilis* 168 (G, H) in the presence of 0 µM (black), 0.3 µM (red), 3 µM (green), and 30 µM (blue) of chalcone **10** (2',3',4'-Trihydroxychalcone). The cells were cultivated in complex media (BHI medium; A, C, E, G) or minimal media (CGXII, B; M9, D, F; Spizizen, H) containing 2% (*w*/*v*) glucose. The cell density was monitored in the Biolector system measuring the backscatter at 620 nm (gain 20).

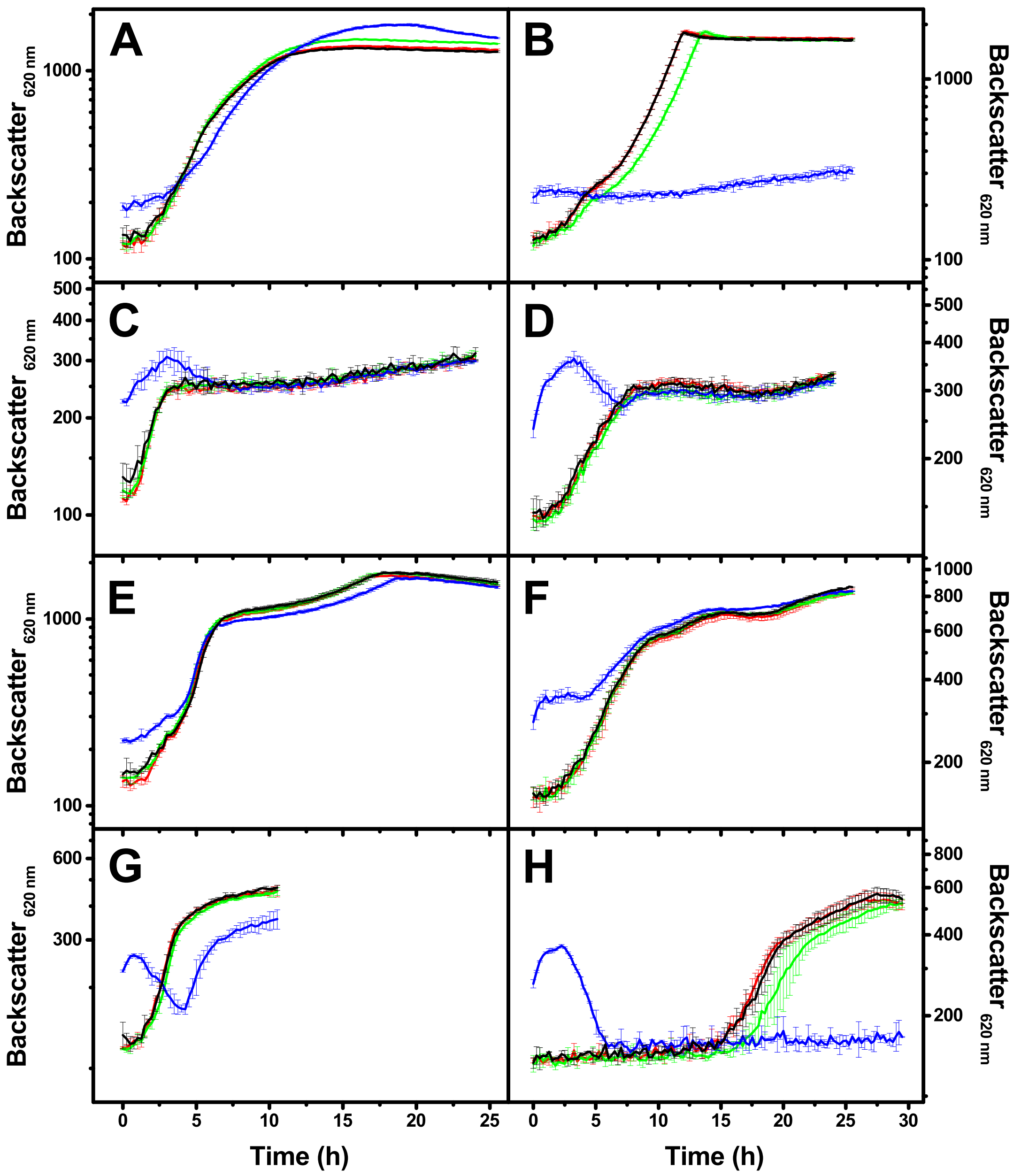

Figure S10: Cultivation of bacteria in the presence of chalcone 5.

Cultivation of *C. glutamicum* ATCC 13032 (A, B), *E. coli* K-12 MG1655 (C, D), *P. putida* KT2440 (E, F), and *B. subtilis* 168 (G, H) in the presence of 0 µM (black), 5.5 µM (red), 55 µM (green), and 550 µM (blue) chalcone **5** (2',5'-Dihydroxychalcone). The cells were cultivated in complex media (BHI medium; A, C, E, G) or minimal media (CGXII, B; M9, D, F; Spizizen, H) containing 2% (*w*/*v*) glucose. The cell density was monitored in the Biolector system measuring the backscatter at 620 nm (gain 20).

Supplemental Tables

Table S1: Parameters of the AFMoC analyses for chalcone ligands in **C_4** PEPC.

Spacing[a]	1.0
σ [a,b]	0.85
q^2 [c,d]	0.63 (0.63)
s_{press} [e,f]	0.10
r^2 [c,g]	0.99 (0.99)
s [e,h]	0.08
F [c,i]	175.6 (175.6)
Components[j]	3
Fraction[k,l]	
C.2	0.01
C.ar	0.37
O.3	0.61
O.2	0.01

[a] In Å.
[b] Half-width of the convolution of the receptor potential fields with the Gaussian function representing the ligand atoms.
[c] Values are given considering only the part of pEC_{50} used in the PLS analysis (pEC_{50}^{PLS}) or considering the total pEC_{50} (values in parentheses).
[d] q^2 = 1 - PRESS/SSD as obtained by "leave-one-out" cross-validation. PRESS equals the sum of squared differences between predicted and experimentally determined binding affinities, SSD is the sum of the squared differences between experimentally determined binding affinities and the mean of the training set binding affinities.
[e] In logarithmic units.
[f] $s_{\text{press}} = \sqrt{(\frac{PRESS}{(n-h-1)})}$ as obtained by "leave-one-out" cross-validation. n equals the number of data points, h is the number of components.
[g] Correlation coefficient.
[h] $s = \sqrt{(\frac{RSS}{n-h-1})}$. RSS equals the sum of squared differences between fitted and experimentally determined binding affinities.
[i] Fisher's F-value.
[j] Number of components.
[k] Fraction of the explained variance due to an interaction field of the given Sybyl atom type.

Table S2: Complete growth data of cultivations of *C. glutamicum* ATCC 13032, *E. coli* K-12 MG1655, *P. putida* KT2440, and *B. subtilis* 168 in the presence of chalcone derivatives.

The bacterial strains were cultured in a microscale cultivation system both in complex and minimal media in the presence of 0.1x, 1x, and 10x the IC_{50} concentration determined for the *F. trinervia* PEP carboxylase. The table indicates whether the final cell density (measured as backscatter at 620 nm) or the growth rate decreased (fbs↓ and µ↓, respectively), or the lag phase was extended (lag↑). All values are given relatively to an untreated control. The colored background of values indicates a deviation of the mean from the control higher than 10% (excluding standard deviation). Effects due to precipitation in the growth media occurring at 10x IC_{50} of *trans*-chalcone and 2'-Hydroxychalcone are indicated by a red background. Abbreviations: StDev, standard deviation; n.d., not determinable.

Table attached as separate file (Table S2.xlxs)

Organism		*C. glutamicum*							
**Inhibitor**	**Media**	**Complex**				**Minimal**			
trans **-Chalcone**	**Concentration**	*8 µM*	*80 µM*		*400 µM*	*8 µM*	*80 µM*	*160 µM*	*400 µM*
	**Final Backscatter**	98,7%	103,8%		106,0%	99,7%	97,0%	98,3%	99,5%
	StDev (±)	2,6%	1,9%		3,0%	2,8%	1,9%	1,3%	2,4%
	**Growth rate**	105,0%	102,9%		102,7%	99,3%	97,1%	92,5%	87,5%
	StDev (±)	3,4%	4,6%		2,6%	2,4%	1,9%	3,9%	1,6%
	**Lag Phase**	100,0%	99,5%		103,7%	100,0%	102,3%	109,9%	113,6%
	StDev (±)	1,3%	1,3%		0,9%	0,0%	0,0%	2,5%	0,0%
**Amb1394996**	**Concentration**	*5 µM*	*50 µM*		500 µM	*5 µM*	*50 µM*	*100 µM*	500 µM
**2'-Hydroxychalcone**	**Final Backscatter**	100,9%	105,4%		111,1%	101,9%	99,6%	101,2%	102,2%
	StDev (±)	2,8%	2,0%		4,1%	1,1%	2,2%	1,3%	1,0%
	**Growth rate**	96,2%	91,5%		101,9%	100,6%	99,6%	100,9%	92,9%
	StDev (±)	5,6%	5,7%		9,3%	1,4%	1,4%	1,7%	1,9%
	**Lag Phase**	98,4%	100,0%		101,1%	98,7%	102,6%	104,2%	124,3%
	StDev (±)	2,0%	2,1%		1,9%	2,2%	3,0%	1,8%	3,8%
**Amb2781549**	**Concentration**	*4 µM*	*40 µM*	*120 µM*	*400 µM*	*4 µM*	*40 µM*	*120 µM*	*400 µM*
**2',4-Dihydroxychalcone**	**Final Backscatter**	100,3%	102,7%	110,6%	111,5%	102,1%	102,6%	101,6%	105,7%
	StDev (±)	1,6%	1,0%	1,9%	0,9%	1,4%	2,0%	2,0%	1,1%
	**Growth rate**	101,0%	99,1%	107,1%	93,9%	99,6%	99,5%	96,7%	81,2%
	StDev (±)	3,3%	3,3%	11,8%	2,9%	2,0%	2,2%	2,5%	2,6%
	**Lag Phase**	93,2%	93,8%	100,5%	118,1%	100,0%	97,3%	107,7%	139,3%
	StDev (±)	2,7%	2,9%	4,7%	3,6%	5,7%	4,5%	2,5%	5,7%
**Amb6356569**	**Concentration**	*4 µM*	*40 µM*		*400 µM*	*4 µM*	*40 µM*		*400 µM*
**2',4'-Dihydroxychalcone**	**Final Backscatter**	101,3%	105,2%		107,4%	102,0%	100,0%		110,5%
	StDev (±)	2,2%	2,3%		2,3%	2,0%	2,2%		2,2%
	**Growth rate**	112,8%	115,1%		91,6%	98,9%	95,5%		65,2%
	StDev (±)	9,9%	11,0%		8,7%	2,6%	3,3%		1,6%
	**Lag Phase**	97,4%	96,2%		116,6%	98,7%	100,0%		169,9%
	StDev (±)	2,2%	0,0%		4,4%	3,6%	2,8%		6,8%
**Amb1486576**	**Concentration**	*5.5 µM*	*55 µM*	*110 µM*	*550 µM*	*5.5 µM*	*55 µM*	*110 µM*	*550 µM*
**2',5'-Dihydroxychalcone**	**Final Backscatter**	102,4%	105,7%	103,1%	118,4%	101,8%	99,7%	101,2%	18,8%
	StDev (±)	1,6%	2,1%	1,9%	1,5%	1,7%	1,8%	1,7%	0,8%
	**Growth rate**	108,7%	106,7%	110,0%	79,0%	94,4%	86,9%	79,5%	n.d.
	StDev (±)	9,7%	10,2%	10,4%	7,4%	1,3%	1,6%	1,4%	n.d.
	**Lag Phase**	98,4%	96,2%	98,4%	120,7%	102,1%	114,0%	133,1%	n.d.
	StDev (±)	1,3%	3,7%	3,4%	2,6%	1,7%	2,8%	2,7%	n.d.
**Amb4477660**	**Concentration**	*4 µM*	*40 µM*		*400 µM*	*4 µM*	*40 µM*		*400 µM*
**2',4',4-Trihydroxychalcone**	**Final Backscatter**	99,8%	98,2%		97,7%	100,1%	101,2%		101,2%
	StDev (±)	1,8%	1,6%		1,0%	1,4%	1,2%		1,3%
	**Growth rate**	99,5%	100,6%		79,8%	101,0%	97,7%		80,5%
	StDev (±)	3,4%	3,2%		2,8%	1,5%	1,5%		1,1%
	**Lag Phase**	99,3%	101,3%		105,3%	98,5%	101,5%		127,6%
	StDev (±)	3,2%	4,7%		1,7%	5,1%	5,8%		5,8%
**Amb4000666**	**Concentration**	*4 µM*	*40 µM*		*400 µM*	*4 µM*	*40 µM*		*400 µM*
**2',4',2-Trihydroxychalcone**	**Final Backscatter**	100,4%	98,3%		109,8%	98,1%	100,9%		94,3%
	StDev (±)	2,7%	2,2%		2,1%	1,8%	2,4%		1,9%
	**Growth rate**	99,1%	98,8%		91,2%	101,6%	98,7%		52,9%
	StDev (±)	3,6%	4,4%		12,8%	1,2%	2,1%		1,9%
	**Lag Phase**	101,6%	106,3%		141,2%	100,0%	103,0%		181,9%
	StDev (±)	2,7%	2,7%		2,7%	3,7%	4,4%		10,3%
**Butein**	**Concentration**	*0.4 µM*	*4 µM*		*40 µM*	*0.4 µM*	*4 µM*		*40 µM*
**2',4',3,4-Tetrahydroxychalcone**	**Final Backscatter**	96,6%	106,6%		108,1%	99,0%	101,1%		103,0%
	StDev (±)	2,3%	2,4%		2,7%	1,1%	2,1%		1,0%
	**Growth rate**	113,5%	133,5%		128,9%	103,0%	96,6%		102,0%
	StDev (±)	11,2%	13,5%		15,0%	3,5%	2,7%		3,2%
	**Lag Phase**	109,9%	95,4%		84,9%	99,3%	98,6%		100,7%
	StDev (±)	4,6%	9,9%		5,8%	1,2%	1,7%		1,7%
**Robtein**	**Concentration**	*0.3 µM*	*3 µM*		*30 µM*	*0.3 µM*	*3 µM*		*30 µM*
**2',4',3,4,5-Pentahydroxychalcone**	**Final Backscatter**	99,6%	101,8%		96,7%	99,9%	96,9%		97,7%
	StDev (±)	3,3%	2,1%		2,6%	2,4%	2,7%		2,5%
	**Growth rate**	104,0%	110,8%		112,2%	104,0%	103,2%		97,3%
	StDev (±)	6,3%	7,4%		10,5%	1,2%	1,6%		1,2%
	**Lag Phase**	98,5%	98,0%		94,5%	98,6%	98,6%		98,6%
	StDev (±)	2,4%	2,3%		2,3%	1,2%	1,2%		1,2%
**Amb5717789**	**Concentration**	*0.3 µM*	*3 µM*		*30 µM*	*0.3 µM*	*3 µM*		*30 µM*
**2',3',4'-Trihydroxychalcone**	**Final Backscatter**	105,4%	108,6%		110,1%	102,6%	103,7%		104,5%
	StDev (±)	5,2%	6,0%		5,6%	1,7%	2,0%		1,6%
	**Growth rate**	106,4%	112,1%		117,7%	104,4%	102,6%		99,3%
	StDev (±)	16,1%	16,8%		20,5%	1,3%	1,5%		2,0%
	**Lag Phase**	100,0%	97,8%		94,9%	97,9%	98,6%		102,1%
	StDev (±)	0,0%	3,8%		2,3%	1,7%	1,7%		1,7%
**Amb6356955**	**Concentration**	*0.3 µM*	*3 µM*		*30 µM*	*0.3 µM*	*3 µM*		*30 µM*
**2',4',6',3,4-Pentahydroxychalcone**	**Final Backscatter**	99,6%	103,2%		102,8%	100,2%	95,1%		96,2%
	StDev (±)	1,6%	0,9%		4,4%	2,5%	1,6%		2,1%
	**Growth rate**	94,6%	104,0%		95,3%	104,4%	105,0%		104,1%
	StDev (±)	12,1%	7,9%		7,6%	2,6%	3,7%		2,5%
	**Lag Phase**	97,0%	97,0%		98,5%	99,3%	98,5%		98,5%
	StDev (±)	2,8%	2,8%		3,9%	4,3%	5,1%		5,1%
**Okanin**	**Concentration**	*0.1 µM*	*1 µM*		*10 µM*	*0.1 µM*	*1 µM*		*10 µM*
**2',3',4',3,4-Pentahydroxychalcone**	**Final Backscatter**	97,0%	93,8%		89,6%	99,6%	97,6%		96,7%
	StDev (±)	3,6%	2,8%		2,7%	1,3%	1,5%		1,2%
	**Growth rate**	108,3%	132,5%		143,8%	101,6%	101,5%		97,9%
	StDev (±)	11,9%	7,8%		9,1%	2,0%	2,2%		1,5%
	**Lag Phase**	96,4%	84,1%		99,3%	99,3%	99,3%		100,7%
	StDev (±)	6,1%	5,0%		6,6%	1,6%	1,6%		1,2%

Organism		*E. coli*					
**Inhibitor**	**Media**	**Complex**			**Minimal**		
trans **-Chalcone**	**Concentration**	*8 µM*	*80 µM*	800 µM	*8 µM*	*80 µM*	800 µM
	**Final Backscatter**	99,0%	97,4%	108,5%	100,1%	95,1%	102,9%
	StDev (±)	5,1%	4,1%	5,6%	3,3%	3,0%	7,3%
	**Growth rate**	104,6%	109,1%	94,7%	97,5%	103,7%	107,4%
	StDev (±)	18,3%	9,2%	9,8%	11,2%	10,9%	18,3%
	**Lag Phase**	102,4%	102,4%	101,2%	99,1%	101,8%	97,8%
	StDev (±)	10,8%	12,0%	11,7%	5,7%	10,5%	12,8%
**Amb1394996**	**Concentration**	*5 µM*	*50 µM*	500 µM	*5 µM*	*50 µM*	500 µM
**2'-Hydroxychalcone**	**Final Backscatter**	100,2%	103,1%	98,1%	100,3%	97,0%	100,7%
	StDev (±)	2,5%	4,1%	3,6%	2,7%	3,1%	3,8%
	**Growth rate**	97,7%	86,6%	n.d.	101,7%	104,8%	n.d.
	StDev (±)	21,2%	15,8%	n.d.	15,0%	15,0%	n.d.
	**Lag Phase**	95,5%	100,0%	n.d.	89,4%	91,6%	n.d.
	StDev (±)	7,8%	5,6%	n.d.	10,0%	6,9%	n.d.
**Amb2781549**	**Concentration**	*4 µM*	*40 µM*	*400 µM*	*4 µM*	*40 µM*	*400 µM*
**2',4-Dihydroxychalcone**	**Final Backscatter**	100,9%	102,1%	98,2%	100,4%	99,1%	100,6%
	StDev (±)	1,7%	2,2%	1,8%	6,2%	4,7%	6,6%
	**Growth rate**	88,3%	95,0%	99,2%	106,5%	95,3%	107,6%
	StDev (±)	20,7%	18,1%	19,4%	16,5%	16,2%	35,7%
	**Lag Phase**	101,4%	102,1%	97,8%	99,3%	100,0%	98,6%
	StDev (±)	4,0%	4,1%	4,2%	0,6%	0,0%	1,2%
**Amb6356569**	**Concentration**	*4 µM*	*40 µM*	*400 µM*	*4 µM*	*40 µM*	*400 µM*
**2',4'-Dihydroxychalcone**	**Final Backscatter**	100,8%	103,5%	100,0%	103,4%	99,3%	101,7%
	StDev (±)	2,7%	2,6%	2,4%	5,5%	4,6%	4,4%
	**Growth rate**	79,6%	97,2%	89,4%	79,0%	77,8%	76,5%
	StDev (±)	20,1%	32,0%	20,3%	19,8%	19,0%	25,4%
	**Lag Phase**	102,2%	101,8%	101,5%	100,7%	99,3%	101,1%
	StDev (±)	1,1%	1,7%	1,3%	1,2%	2,0%	1,1%
**Amb1486576**	**Concentration**	*5.5 µM*	*55 µM*	*550 µM*	*5.5 µM*	*55 µM*	*550 µM*
**2',5'-Dihydroxychalcone**	**Final Backscatter**	101,6%	101,4%	102,6%	99,8%	99,1%	96,4%
	StDev (±)	4,1%	3,7%	3,0%	1,0%	4,3%	2,3%
	**Growth rate**	101,5%	102,6%	n.d.	108,5%	122,0%	n.d.
	StDev (±)	59,6%	51,2%	n.d.	29,6%	45,3%	n.d.
	**Lag Phase**	104,7%	103,1%	117,2%	101,0%	105,9%	103,0%
	StDev (±)	36,0%	35,1%	22,7%	11,3%	10,3%	10,8%
**Amb4477660**	**Concentration**	*4 µM*	*40 µM*	*400 µM*	*4 µM*	*40 µM*	*400 µM*
**2',4',4-Trihydroxychalcone**	**Final Backscatter**	98,8%	99,5%	95,0%	100,5%	98,8%	97,7%
	StDev (±)	2,3%	1,8%	3,3%	2,5%	2,9%	2,3%
	**Growth rate**	96,0%	99,1%	89,8%	100,8%	107,2%	96,4%
	StDev (±)	7,9%	9,1%	7,0%	19,5%	11,3%	13,7%
	**Lag Phase**	100,0%	100,0%	107,4%	101,8%	103,5%	101,8%
	StDev (±)	0,0%	0,0%	6,4%	6,1%	8,1%	6,1%
**Amb4000666**	**Concentration**	*4 µM*	*40 µM*	*400 µM*	*4 µM*	*40 µM*	*400 µM*
**2',4',2-Trihydroxychalcone**	**Final Backscatter**	97,2%	97,3%	93,0%	98,2%	97,2%	97,4%
	StDev (±)	4,1%	3,5%	3,9%	1,1%	1,5%	3,3%
	**Growth rate**	93,3%	90,7%	104,3%	99,2%	104,8%	103,3%
	StDev (±)	4,8%	5,0%	6,0%	13,6%	14,4%	15,1%
	**Lag Phase**	107,4%	111,1%	118,5%	88,9%	99,0%	99,3%
	StDev (±)	13,5%	12,3%	14,6%	18,6%	1,3%	1,3%
**Butein**	**Concentration**	*0.4 µM*	*4 µM*	*40 µM*	*0.4 µM*	*4 µM*	*40 µM*
**2',4',3,4-Tetrahydroxychalcone**	**Final Backscatter**	98,9%	101,6%	95,7%	92,9%	94,1%	96,6%
	StDev (±)	2,1%	3,7%	2,5%	2,8%	1,8%	1,7%
	**Growth rate**	101,8%	95,0%	89,4%	83,9%	86,4%	85,3%
	StDev (±)	26,7%	19,6%	20,3%	8,8%	4,3%	5,0%
	**Lag Phase**	103,3%	96,7%	100,0%	96,2%	98,1%	98,1%
	StDev (±)	11,8%	11,2%	10,0%	4,6%	3,3%	3,3%
**Robtein**	**Concentration**	*0.3 µM*	*3 µM*	*30 µM*	*0.3 µM*	*3 µM*	*30 µM*
**2',4',3,4,5-Pentahydroxychalcone**	**Final Backscatter**	100,0%	99,7%	98,2%	100,4%	100,9%	101,5%
	StDev (±)	2,7%	3,9%	4,0%	3,3%	1,8%	3,2%
	**Growth rate**	103,1%	95,4%	87,6%	105,8%	110,4%	109,6%
	StDev (±)	22,8%	15,3%	19,0%	14,3%	8,7%	12,1%
	**Lag Phase**	103,4%	103,4%	110,3%	100,0%	98,2%	96,4%
	StDev (±)	6,2%	12,0%	8,9%	13,8%	11,4%	13,3%
**Amb5717789**	**Concentration**	*0.3 µM*	*3 µM*	*30 µM*	*0.3 µM*	*3 µM*	*30 µM*
**2',3',4'-Trihydroxychalcone**	**Final Backscatter**	106,5%	106,2%	98,9%	105,4%	104,6%	108,6%
	StDev (±)	4,2%	7,3%	2,2%	1,8%	2,1%	4,4%
	**Growth rate**	109,4%	99,8%	104,1%	96,3%	92,0%	99,2%
	StDev (±)	17,2%	9,4%	21,5%	20,8%	19,7%	24,3%
	**Lag Phase**	92,1%	94,8%	118,4%	92,1%	90,6%	92,1%
	StDev (±)	6,2%	9,0%	21,5%	4,2%	4,6%	4,2%
**Amb6356955**	**Concentration**	*0.3 µM*	*3 µM*	*30 µM*	*0.3 µM*	*3 µM*	*30 µM*
**2',4',6',3,4-Pentahydroxychalcone**	**Final Backscatter**	98,0%	98,1%	96,9%	98,0%	98,7%	97,9%
	StDev (±)	3,0%	2,2%	2,7%	0,9%	3,2%	1,3%
	**Growth rate**	104,2%	106,2%	103,9%	107,0%	109,2%	107,2%
	StDev (±)	11,9%	4,2%	2,7%	8,3%	9,4%	8,0%
	**Lag Phase**	107,1%	107,1%	107,1%	103,4%	101,7%	103,4%
	StDev (±)	6,6%	6,6%	6,6%	22,3%	23,6%	22,3%
**Okanin**	**Concentration**	*0.1 µM*	*1 µM*	*10 µM*	*0.1 µM*	*1 µM*	*10 µM*
**2',3',4',3,4-Pentahydroxychalcone**	**Final Backscatter**	98,5%	100,0%	97,9%	99,7%	98,5%	100,9%
	StDev (±)	4,6%	4,2%	4,6%	3,3%	3,0%	3,1%
	**Growth rate**	105,5%	100,2%	111,4%	99,8%	100,1%	103,6%
	StDev (±)	12,9%	12,8%	22,8%	11,8%	12,8%	12,4%
	**Lag Phase**	108,5%	102,8%	111,4%	99,0%	102,0%	95,1%
	StDev (±)	7,3%	9,9%	10,2%	11,3%	11,6%	11,1%

Organism		*P. putida*					
**Inhibitor**	**Media**	**Complex**			**Minimal**		
trans **-Chalcone**	**Concentration**	*8 µM*	*80 µM*	*400 µM*	8 µM	80 µM	*400 µM*
	**Final Backscatter**	95,3%	96,7%	97,3%	94,4%	94,2%	100,2%
	StDev (±)	0,6%	1,1%	2,0%	3,8%	0,4%	8,8%
	**Growth rate**	98,4%	100,5%	102,6%	112,4%	112,8%	119,2%
	StDev (±)	4,0%	4,2%	4,0%	3,4%	3,6%	5,0%
	**Lag Phase**	100,6%	101,8%	103,0%	98,9%	99,3%	98,1%
	StDev (±)	2,1%	4,7%	2,1%	3,0%	3,8%	3,4%
**Amb1394996**	**Concentration**	*5 µM*	*50 µM*	500 µM	*5 µM*	*50 µM*	500 µM
**2'-Hydroxychalcone**	**Final Backscatter**	98,0%	100,9%	102,2%	96,6%	98,0%	110,9%
	StDev (±)	2,5%	2,1%	2,2%	2,7%	1,9%	3,4%
	**Growth rate**	103,2%	102,4%	188,9%	96,0%	99,3%	153,4%
	StDev (±)	3,0%	3,3%	19,1%	5,6%	6,5%	15,1%
	**Lag Phase**	101,0%	102,6%	110,8%	100,0%	100,6%	100,0%
	StDev (±)	0,9%	0,9%	0,0%	1,4%	1,0%	1,4%
**Amb2781549**	**Concentration**	*4 µM*	*40 µM*	*400 µM*	*4 µM*	*40 µM*	*400 µM*
**2',4-Dihydroxychalcone**	**Final Backscatter**	99,6%	102,9%	99,5%	97,4%	100,6%	110,5%
	StDev (±)	1,9%	2,3%	2,1%	1,8%	1,7%	1,7%
	**Growth rate**	103,0%	105,6%	107,7%	95,3%	97,1%	106,5%
	StDev (±)	6,1%	5,6%	5,9%	3,0%	7,9%	5,7%
	**Lag Phase**	97,7%	98,5%	98,5%	102,8%	101,6%	103,2%
	StDev (±)	0,0%	1,3%	1,3%	3,0%	4,0%	2,9%
**Amb6356569**	**Concentration**	*4 µM*	*40 µM*	*400 µM*	*4 µM*	*40 µM*	*400 µM*
**2',4'-Dihydroxychalcone**	**Final Backscatter**	97,8%	101,5%	100,5%	102,5%	102,3%	107,4%
	StDev (±)	2,7%	2,2%	3,3%	13,8%	12,6%	12,0%
	**Growth rate**	100,6%	100,1%	101,3%	94,1%	98,3%	93,9%
	StDev (±)	7,5%	6,9%	7,1%	9,7%	10,8%	10,3%
	**Lag Phase**	105,0%	106,6%	123,2%	108,7%	108,3%	97,7%
	StDev (±)	12,1%	8,3%	8,0%	9,2%	9,2%	18,1%
**Amb1486576**	**Concentration**	*5.5 µM*	*55 µM*	*550 µM*	*5.5 µM*	*55 µM*	*550 µM*
**2',5'-Dihydroxychalcone**	**Final Backscatter**	95,2%	98,5%	94,4%	96,1%	95,5%	96,7%
	StDev (±)	2,6%	3,2%	2,9%	2,2%	0,9%	0,4%
	**Growth rate**	106,1%	104,9%	118,4%	94,9%	99,4%	101,0%
	StDev (±)	6,5%	7,1%	7,1%	17,0%	18,6%	16,3%
	**Lag Phase**	100,0%	99,1%	105,0%	100,7%	101,0%	98,0%
	StDev (±)	3,0%	6,9%	5,3%	0,6%	0,0%	1,7%
**Amb4477660**	**Concentration**	*4 µM*	*40 µM*	*400 µM*	*4 µM*	*40 µM*	*400 µM*
**2',4',4-Trihydroxychalcone**	**Final Backscatter**	97,7%	97,2%	99,9%	99,0%	102,6%	117,1%
	StDev (±)	0,7%	1,3%	1,5%	4,2%	3,1%	3,6%
	**Growth rate**	103,8%	105,6%	100,0%	104,2%	106,1%	104,0%
	StDev (±)	3,1%	3,1%	2,3%	7,8%	4,6%	4,3%
	**Lag Phase**	99,4%	101,7%	107,3%	103,3%	105,6%	107,8%
	StDev (±)	1,9%	2,4%	2,1%	3,3%	1,9%	1,9%
**Amb4000666**	**Concentration**	*4 µM*	*40 µM*	*400 µM*	*4 µM*	*40 µM*	*400 µM*
**2',4',2-Trihydroxychalcone**	**Final Backscatter**	99,1%	95,3%	99,9%	101,3%	100,0%	99,8%
	StDev (±)	2,3%	2,5%	2,1%	2,8%	3,2%	3,1%
	**Growth rate**	100,0%	101,0%	99,6%	98,3%	94,3%	106,1%
	StDev (±)	6,8%	6,7%	6,4%	7,4%	7,4%	8,3%
	**Lag Phase**	100,0%	100,0%	100,0%	111,6%	101,6%	95,6%
	StDev (±)	0,0%	0,0%	0,0%	5,6%	10,2%	15,2%
**Butein**	**Concentration**	*0.4 µM*	*4 µM*	*40 µM*	*0.4 µM*	*4 µM*	*40 µM*
**2',4',3,4-Tetrahydroxychalcone**	**Final Backscatter**	94,7%	97,0%	94,9%	87,0%	89,8%	94,8%
	StDev (±)	1,9%	2,1%	2,1%	2,8%	1,3%	1,0%
	**Growth rate**	90,8%	97,4%	95,3%	83,7%	89,0%	90,0%
	StDev (±)	11,0%	10,0%	7,9%	6,4%	9,9%	9,6%
	**Lag Phase**	92,6%	93,2%	96,3%	99,6%	99,6%	98,5%
	StDev (±)	0,0%	1,1%	0,0%	0,6%	1,3%	0,6%
**Robtein**	**Concentration**	*0.3 µM*	*3 µM*	*30 µM*	*0.3 µM*	*3 µM*	*30 µM*
**2',4',3,4,5-Pentahydroxychalcone**	**Final Backscatter**	94,7%	96,9%	93,0%	93,1%	96,5%	98,3%
	StDev (±)	2,0%	2,6%	3,1%	4,5%	2,9%	2,4%
	**Growth rate**	99,4%	100,7%	101,8%	108,6%	108,4%	108,0%
	StDev (±)	2,7%	2,3%	3,3%	9,7%	8,1%	12,3%
	**Lag Phase**	101,2%	102,4%	99,4%	99,0%	99,3%	99,7%
	StDev (±)	1,5%	1,1%	1,5%	1,3%	0,8%	1,2%
**Amb5717789**	**Concentration**	*0.3 µM*	*3 µM*	*30 µM*	*0.3 µM*	*3 µM*	*30 µM*
**2',3',4'-Trihydroxychalcone**	**Final Backscatter**	103,1%	102,5%	105,2%	101,3%	100,6%	102,7%
	StDev (±)	6,6%	6,7%	6,6%	4,5%	3,5%	1,4%
	**Growth rate**	114,1%	103,2%	105,1%	94,8%	96,0%	91,3%
	StDev (±)	8,7%	9,0%	7,6%	5,6%	8,4%	6,1%
	**Lag Phase**	103,9%	100,9%	107,3%	100,0%	101,5%	99,2%
	StDev (±)	10,8%	11,1%	10,0%	1,1%	2,9%	1,7%
**Amb6356955**	**Concentration**	*0.3 µM*	*3 µM*	*30 µM*	*0.3 µM*	*3 µM*	*30 µM*
**2',4',6',3,4-Pentahydroxychalcone**	**Final Backscatter**	92,6%	96,8%	92,5%	93,9%	94,9%	99,0%
	StDev (±)	1,2%	2,5%	2,0%	2,1%	1,4%	1,4%
	**Growth rate**	107,9%	106,4%	105,3%	96,8%	98,7%	95,2%
	StDev (±)	2,0%	2,0%	2,4%	13,7%	5,6%	6,5%
	**Lag Phase**	95,5%	97,5%	97,0%	99,3%	98,9%	98,9%
	StDev (±)	3,7%	4,5%	4,3%	0,6%	0,0%	0,0%
**Okanin**	**Concentration**	*0.1 µM*	*1 µM*	*10 µM*	*0.1 µM*	*1 µM*	*10 µM*
**2',3',4',3,4-Pentahydroxychalcone**	**Final Backscatter**	97,8%	98,2%	98,6%	92,5%	93,6%	99,3%
	StDev (±)	1,3%	1,2%	2,6%	5,4%	5,6%	6,3%
	**Growth rate**	104,5%	103,0%	99,7%	105,1%	103,9%	100,1%
	StDev (±)	7,1%	6,0%	6,8%	4,5%	4,5%	3,5%
	**Lag Phase**	103,3%	103,9%	105,9%	108,8%	106,1%	100,0%
	StDev (±)	2,3%	5,2%	3,4%	6,6%	6,1%	6,4%

Organism		*B. subtilis*							
**Inhibitor**	**Media**	**Complex**				**Minimal**			
trans **-Chalcone**	**Concentration**	*8 µM*	*80 µM*		800 µM	*8 µM*	*80 µM*	*160 µM*	800 µM
	**Final Backscatter**	95,4%	99,0%		102,9%	89,6%	88,8%	89,5%	83,6%
	StDev (±)	2,0%	3,9%		3,0%	9,0%	2,1%	3,9%	8,7%
	**Growth rate**	114,9%	119,3%		130,3%	117,6%	104,9%	97,3%	93,3%
	StDev (±)	5,8%	2,5%		9,5%	11,3%	8,9%	8,9%	7,2%
	**Lag Phase**	103,5%	103,5%		101,2%	111,6%	109,9%	110,6%	121,3%
	StDev (±)	4,6%	4,6%		5,4%	11,2%	1,2%	0,0%	3,3%
**Amb1394996**	**Concentration**	*5 µM*	*50 µM*	*100 µM*	500 µM	*5 µM*	*50 µM*	*100 µM*	500 µM
**2'-Hydroxychalcone**	**Final Backscatter**	96,4%	97,6%	112,0%	75,6%	97,9%	95,5%	100,6%	70,0%
	StDev (±)	3,2%	2,8%	6,0%	2,0%	4,1%	2,4%	3,8%	6,7%
	**Growth rate**	91,2%	97,7%	117,8%	n.d.	96,8%	112,9%	134,1%	98,7%
	StDev (±)	15,4%	14,8%	15,7%	n.d.	6,2%	7,9%	12,0%	7,6%
	**Lag Phase**	89,8%	100,0%	95,4%	n.d.	99,5%	108,5%	107,8%	143,1%
	StDev (±)	66,0%	13,3%	11,1%	n.d.	3,4%	4,3%	1,2%	7,1%
**Amb2781549**	**Concentration**	*4 µM*	*40 µM*	*120 µM*	*400 µM*	*4 µM*	*40 µM*	*120 µM*	*400 µM*
**2',4-Dihydroxychalcone**	**Final Backscatter**	95,2%	97,1%	101,9%	91,6%	101,3%	99,1%	100,4%	101,9%
	StDev (±)	2,4%	3,4%	4,1%	2,4%	4,2%	4,0%	2,7%	5,0%
	**Growth rate**	99,5%	107,5%	113,7%	92,8%	129,9%	119,1%	114,6%	112,7%
	StDev (±)	12,4%	7,9%	8,1%	8,7%	6,1%	14,4%	13,8%	6,5%
	**Lag Phase**	105,7%	105,7%	125,5%	151,3%	99,0%	101,0%	111,3%	151,4%
	StDev (±)	7,2%	7,2%	15,7%	12,4%	18,6%	20,0%	2,5%	49,7%
**Amb6356569**	**Concentration**	*4 µM*	*40 µM*		*400 µM*	*4 µM*	*40 µM*		*400 µM*
**2',4'-Dihydroxychalcone**	**Final Backscatter**	92,2%	94,3%		81,5%	96,6%	95,7%		77,3%
	StDev (±)	2,7%	2,9%		2,2%	7,7%	6,2%		5,2%
	**Growth rate**	127,8%	97,4%		92,7%	91,5%	108,2%		75,2%
	StDev (±)	27,4%	19,6%		18,2%	16,1%	22,2%		13,7%
	**Lag Phase**	107,7%	107,7%		166,5%	97,7%	100,0%		129,1%
	StDev (±)	0,0%	0,0%		11,7%	2,0%	2,8%		2,2%
**Amb1486576**	**Concentration**	*5.5 µM*	*55 µM*	*110 µM*	*550 µM*	*5.5 µM*	*55 µM*	*110 µM*	*550 µM*
**2',5'-Dihydroxychalcone**	**Final Backscatter**	97,2%	96,2%	98,0%	76,3%	95,2%	96,4%	102,5%	30,5%
	StDev (±)	2,9%	2,9%	3,6%	6,2%	5,1%	4,8%	2,2%	1,1%
	**Growth rate**	98,8%	108,5%	107,6%	n.d.	100,1%	107,0%	109,9%	n.d.
	StDev (±)	17,2%	11,2%	12,0%	n.d.	23,9%	22,1%	8,7%	n.d.
	**Lag Phase**	95,5%	102,3%	109,3%	177,1%	99,4%	103,5%	108,5%	n.d.
	StDev (±)	3,7%	7,9%	13,8%	9,7%	5,4%	3,2%	0,0%	n.d.
**Amb4477660**	**Concentration**	*4 µM*	*40 µM*		*400 µM*	*4 µM*	*40 µM*		*400 µM*
**2',4',4-Trihydroxychalcone**	**Final Backscatter**	97,3%	99,9%		91,1%	100,2%	99,7%		100,9%
	StDev (±)	20,6%	22,6%		21,3%	3,4%	2,7%		1,6%
	**Growth rate**	106,6%	107,4%		108,6%	102,9%	99,0%		88,2%
	StDev (±)	20,4%	17,9%		17,9%	8,6%	11,8%		7,0%
	**Lag Phase**	100,0%	102,6%		128,1%	98,9%	102,2%		116,3%
	StDev (±)	10,8%	9,0%		20,3%	2,6%	2,7%		4,4%
**Amb4000666**	**Concentration**	*4 µM*	*40 µM*		*400 µM*	*4 µM*	*40 µM*		*400 µM*
**2',4',2-Trihydroxychalcone**	**Final Backscatter**	99,9%	102,3%		98,3%	98,8%	98,5%		95,3%
	StDev (±)	24,3%	25,6%		30,1%	2,1%	1,9%		1,9%
	**Growth rate**	95,9%	104,5%		92,5%	106,7%	107,3%		70,4%
	StDev (±)	5,1%	6,7%		5,5%	15,6%	9,2%		6,4%
	**Lag Phase**	100,0%	107,1%		185,2%	104,9%	106,1%		143,2%
	StDev (±)	18,9%	16,9%		29,2%	3,6%	2,5%		5,4%
**Butein**	**Concentration**	*0.4 µM*	*4 µM*		*40 µM*	*0.4 µM*	*4 µM*		*40 µM*
**2',4',3,4-Tetrahydroxychalcone**	**Final Backscatter**	98,1%	100,1%		98,9%	85,1%	92,1%		94,2%
	StDev (±)	4,6%	5,1%		4,6%	3,0%	3,3%		7,6%
	**Growth rate**	98,2%	110,9%		109,4%	106,4%	104,9%		95,7%
	StDev (±)	17,1%	18,5%		15,1%	19,5%	26,4%		23,7%
	**Lag Phase**	98,3%	86,5%		105,1%	107,5%	110,3%		90,4%
	StDev (±)	17,9%	12,2%		22,3%	30,3%	35,2%		30,6%
**Robtein**	**Concentration**	*0.3 µM*	*3 µM*		*30 µM*	*0.3 µM*	*3 µM*		*30 µM*
**2',4',3,4,5-Pentahydroxychalcone**	**Final Backscatter**	100,3%	99,7%		98,6%	104,2%	103,1%		106,9%
	StDev (±)	1,4%	2,2%		1,7%	4,5%	5,3%		5,9%
	**Growth rate**	110,9%	111,5%		104,5%	99,6%	100,9%		90,7%
	StDev (±)	13,0%	10,9%		12,9%	15,6%	13,2%		11,9%
	**Lag Phase**	100,0%	100,0%		97,4%	91,7%	98,8%		94,1%
	StDev (±)	0,0%	0,0%		4,4%	2,1%	4,2%		2,6%
**Amb5717789**	**Concentration**	*0.3 µM*	*3 µM*		*30 µM*	*0.3 µM*	*3 µM*		*30 µM*
**2',3',4'-Trihydroxychalcone**	**Final Backscatter**	101,5%	101,0%		103,0%	108,3%	109,8%		106,3%
	StDev (±)	3,1%	2,6%		3,5%	24,9%	18,9%		19,1%
	**Growth rate**	106,4%	108,9%		112,4%	88,5%	94,4%		90,6%
	StDev (±)	8,9%	16,9%		9,5%	9,4%	6,3%		1,6%
	**Lag Phase**	100,0%	104,2%		97,9%	98,3%	96,6%		99,3%
	StDev (±)	6,2%	7,4%		7,1%	12,3%	7,9%		8,8%
**Amb6356955**	**Concentration**	*0.3 µM*	*3 µM*		*30 µM*	*0.3 µM*	*3 µM*		*30 µM*
**2',4',6',3,4-Pentahydroxychalcone**	**Final Backscatter**	97,4%	96,5%		97,1%	97,2%	98,7%		102,8%
	StDev (±)	1,4%	1,1%		3,0%	1,7%	1,1%		1,5%
	**Growth rate**	105,8%	106,3%		97,8%	113,7%	107,0%		103,0%
	StDev (±)	11,3%	15,1%		13,0%	5,7%	5,9%		6,8%
	**Lag Phase**	100,0%	100,0%		100,0%	100,0%	101,1%		100,0%
	StDev (±)	7,2%	7,2%		7,2%	2,8%	2,8%		2,8%
**Okanin**	**Concentration**	*0.1 µM*	*1 µM*		*10 µM*	*0.1 µM*	*1 µM*		*10 µM*
**2',3',4',3,4-Pentahydroxychalcone**	**Final Backscatter**	93,5%	93,9%		90,6%	102,0%	98,6%		104,4%
	StDev (±)	3,3%	3,6%		3,3%	11,4%	10,5%		10,3%
	**Growth rate**	96,7%	104,1%		109,3%	123,1%	113,2%		115,0%
	StDev (±)	6,1%	8,0%		12,3%	10,1%	9,4%		10,1%
	**Lag Phase**	110,6%	102,7%		94,7%	98,2%	100,6%		100,0%
	StDev (±)	6,4%	2,2%		8,2%	7,5%	7,3%		6,6%

Table S3: Influence of okanin on maximal rubisco carboxylation rate (V_{cmax}), maximal electron transport rate (J_{max}) and mitochondrial respiration (R_d) of *B. napus* estimated from *in planta* gas exchange measurements. Parameter estimates of the Farquhar-vanCaemmerer-Berry-model of the minimum reduced model are given. P-values indicate significance of the F-test comparing reduced and full models. Values in red are final parameter estimates that are valid for both measurement days due to a lack of statistical significance compared with the full model (n=4).

	parameter	before treatment	one day after treatment	*p*
untreated	V_{cmax}	74.4 (1.82)	94.5 (2.04)	0.001
	J_{max}	136.6 (2.23)	159.0 (2.20)	<0.001
	R_d	0.7 (0.35)		1
$control_{DMSO}$	V_{cmax}	162.8 (26.83)	90.9 (4.82)	<0.001
	J_{max}	158.7 (4.79)		0.453
	R_d	6.3 (0.93)	1.0 (0.90)	<0.001
okanin	V_{cmax}	81.5 (3.33)		
	J_{max}	144.2 (3.77)		0.2998
	R_d	0.9 (0.66)		
